# Nanoscale Oxygenous Heterogeneity in FePC Glass for Highly Efficient and Reusable Catalytic Performance

**DOI:** 10.1002/advs.202304045

**Published:** 2023-09-21

**Authors:** Qi Chen, Lingyu Guo, Haoxiang Di, Zhigang Qi, Zhaoxuan Wang, Ziqi Song, Laichang Zhang, Lina Hu, Weimin Wang

**Affiliations:** ^1^ Key Laboratory for Liquid‐Solid Structural Evolution and Processing of Materials (Ministry of Education) School of Materials Science and Engineering Shandong University Jinan 250061 China; ^2^ School of Transportation and Logistics Engineering Wuhan University of Technology Wuhan 430063 China; ^3^ School of Chemical Engineering and Light Industry Guangdong University of Technology Guangzhou 510006 China; ^4^ School of Engineering Edith Cowan University 270 Joondalup Drive, Joondalup Perth WA 6027 Australia

**Keywords:** chemical heterogeneous, energy barrier, hierarchical gradient, nanoglass, oxygenous

## Abstract

Metallic glass, with its unique disordered atomic structure and high density of low‐coordination sites, is regarded as the most competitive new catalyst for environmental catalysis. However, the efficiency and stability of metallic glass catalysts are often affected by their atomic configuration. Thus, the design and regulation of the nanoscale structure of metallic glasses to improve their catalytic efficiency and stability remains a challenge. Herein, a non‐noble component, Fe_75_P_15_C_10_ amorphous ribbon, is used as a precursor to fabricate a hierarchical gradient catalyst with nanoscale heterogeneous and oxygenous amorphous structure by simple annealing and acid‐immersing. The resulting catalyst offers an ultrahigh catalytic ability of kSA^•^C0 = 3101 mg m^−2^ min^−1^ and excellent reusability of 39 times without efficiency decay in dye wastewater degradation. Theoretical calculations indicate that the excellent catalytic performance of the catalyst can be attributed to its unique heterogeneous nanoglass structure, which induces oxygen atoms. Compared to the FePC structure, the FeP/FePCO structure exhibits strong charge transferability, and the energy barrier of the rate‐determining steps of the conversion of S_2_O_8_^2−^ to SO_4_^−•^ is reduced from 2.52 to 0.97 eV. This study reveals that a heterogeneous nanoglass structure is a new strategy for obtaining high catalytic performance.

## Graphical abstract

A hierarchical gradient catalyst with a nanoscale heterogeneous and oxygenous amorphous structure is fabricated by simple annealing and acid immersion using a non‐noble metal component Fe_75_P_15_C_10_ amorphous ribbon as a precursor; the catalyst afforded ultrahigh catalytic ability and reusability in dye wastewater degradation. Theoretical calculations confirmes that the hierarchical‐gradient catalyst structure exhibits strong charge transferability, and the energy barrier for the rate‐determining steps in converting of S_2_O_8_^2−^ to SO_4_^−•^ is very low.

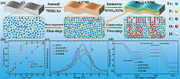

## 1 Introduction

Metallic glass, also known as an amorphous alloy, has become a popular topic in the research of metallic materials because of its microstructure and the existence of ordered and disordered opposites between crystal materials.^[^ ^1^ ^]^ Several important studies have been performed on short‐range ordered structures, such as the coordination number, bond length, electron orbital hybridization, and density distribution of electron states.^[^ ^2^, ^3^ ^]^ Meanwhile, the long‐range disordered atomic arrangement in metallic glass results in a homogeneous distribution of isotropic structures, effectively avoiding structural defects such as grain boundaries, dislocations, and crystal distortion.^[^ ^4^ ^]^ Moreover, metallic glasses exhibit several advanced structures and intriguing functions owing to their unique physicochemical properties, such as unique soft magnetism,^[^ ^4^ ^]^ excellent corrosion resistance,^[^ ^5^ ^]^ high strength,^[^ ^6^ ^]^ and elasticity.^[^ ^7^ ^]^ However, owing to their structural instability,^[^ ^8^ ^]^ easy relaxation and aging characteristics,^[^ ^9^ ^]^ they can only be used in limited applications such as, large transformers, turbine blades, and golf drivers. Thus, developing new practical industrial applications for metallic glasses is urgently needed.

In recent years, metallic glasses have demonstrated significant potential as catalysts in energy and environmental applications. Several studies have shown that metallic glasses have considerable potential for practical catalytic applications because of their unsaturated atomic coordination,^[^ ^2^ ^]^ and homogeneous and isotropic characteristics, which provide abundant catalytically active sites.^[^ ^10^ ^]^ Recent reports have demonstrated that Fe_50_Ni_30_P_13_C_7_ metallic glass ribbons can be used as efficient bifunctional electrode materials for water electro‐splitting in an alkaline medium.^[^ ^11^ ^]^ In another typical example, Fe_83_Si_2_B_11_P_3_C_1_ metallic glass ribbons can function as environmental catalysts with ultralong reusability and a superior catalytic rate owing to their intrinsic isotropy and chemical heterogeneity.^[^ ^12^ ^]^ Meanwhile, annealing‐induced nanocrystals and matrices in (Fe_73.5_Si_13.5_B_9_Nb_3_Cu_1_)_91.5_Ni_8.5_ and Fe_82.65_Si_4_B_12_Cu_1.35_ metallic glass ribbons lead to a galvanic cell effect in the degradation reaction, which can accelerate electron transfer and result in a higher degradation ability than pure metallic glass alloys.^[^ ^13^, ^14^ ^]^ However, recent studies have demonstrated that increased degradation efficiency decreases reusability and vice versa.^[^ ^15^, ^16^ ^]^ Thus, designing and fabricating metallic glass with high degradation ability and strong reusability remain challenging.

To compensate for these defects of metallic glass, researchers have developed a new type of metallic glass with unique structural characteristics, known as nanostructured metallic glass or nanoglass because of its nanometer‐sized heterogeneous structure.^[^ ^17^, ^18^, ^19^ ^]^ The structural model considers that nanostructured metallic glass is composed of amorphous nanometer‐sized grains connected by an amorphous interface (glass/glass interface). The width of the glass/glass interface is only several nanometers, with a locally reduced density relative to the adjacent grains, which results in significant structural heterogeneity and low coordination.^[^ ^19^, ^20^ ^]^ Therefore, the chemical composition and fabrication process of nanostructured metallic glasses can be modified to improve their performance. Recently, some researchers have fabricated Ni–P nanostructured metallic glass with a high‐energy‐state heterogeneous structure, which shows excellent catalytic performance in water‐splitting reactions compared to traditional metallic glass with the same composition.^[^ ^21^ ^]^ However, only a few reports are currently available on the catalytic performance of nanostructured metallic glass.^[^ ^22^ ^]^

Herein, Inspired by the nanostructured metallic glass and its unique chemically heterogeneous glassy structure, a traditional metallic glass ribbon with an atomic composition of Fe_75_P_15_C_10_ was used as a precursor alloy to fabricate a high‐efficiency hierarchical gradient catalyst for the catalytic degradation of dye wastewater. We initially used a simple annealing process to achieve an internal rearrangement of the ribbons. Then, chemically heterogeneous glassy structures were self‐constructed at the nanoscale during the acid treatment process, which played a crucial role in the high degradation capacity and strong reusability. Moreover, density functional theory (DFT) calculations revealed that these unique heterogeneous nanoglass structures, which induce oxygen atoms, are the origin of the high catalytic degradation activity. This study uncovered a new strategy for designing non‐noble nanostructured metallic glass catalysts and revealed that a chemically heterogeneous glassy structure is a new mechanism for obtaining high catalytic degradation performance.

## 2 Results and Discussion

### 2.1 Fabrication and Characterization

**Figure 1:**
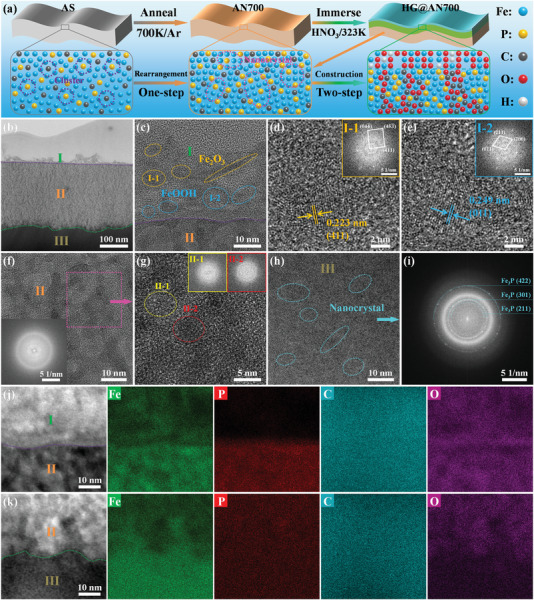
Fabrication and characterization of the hierarchical‐gradient catalyst. a) Schematic illustration of the fabrication process of HG@AN700. b) TEM image of the cross‐sectional structure. c–e) Zoomed selected oxide layer (region I) of (b), showing the nanocrystal structure of Fe_2_O_3_ and FeOOH, and the corresponding FFT patterns are shown in the top right (d) and (e). f) HRTEM image of transition layer (region II) of (b), the corresponding FFT pattern is shown in the lower left, and g) the image consists of two different regions: an interfacial region II−1 and a nanoscaled grain region II−2, the corresponding FFT patterns of the two regions are shown in the top right g,h) HRTEM image of the matrix (region III) of (b), showing the nanocrystal structure of Fe_3_P, and the corresponding FFT pattern is shown in i–k) HAADF‐STEM images of the cross‐sectional structure of original HG@AN700 ribbons; and elemental mapping results of Fe, P, C, and O.

**Figure** **1**a shows a schematic of the evolution of the microstructure during the fabrication of the hierarchical‐gradient catalyst (HG@AN700) with Fe_75_P_15_C_10_ (AS) ribbon as a precursor alloy. The amorphous structure and ultrasmooth surface of the AS ribbon were confirmed by differential scanning calorimetry (DSC)/X‐ray diffraction (XRD) and scanning electron microscope (SEM) (Figures S1 and S2a, Supporting Information). After annealing and acid immersion, the XRD patterns of the AN700 and HG@AN700 ribbons still showed the characteristic peaks of an amorphous structure, but the ultrasmooth surface morphology evolved into a micro‐granular morphology (Figures S1b and S2b,c, Supporting Information). The SEM micrographs of the cross‐sectional structures of various ribbons show that AS and AN700 exhibit a homogeneous composition distribution, whereas HG@AN700 exhibits hierarchical gradient changes in the composition (Figure S3, Supporting Information). To demonstrate the structural evolution clearly, transmission electron microscope (TEM) was performed. The HRTEM image in Figure S4 (Supporting Information) shows that the AS ribbon exhibits a homogeneous amorphous structure, and the corresponding fast Fourier transform (FFT) pattern consists only of a typical diffraction halo. A TEM image of the cross‐sectional structure of the HG@AN700 ribbon is shown in Figure 1b. Notably, the presence of a region is hierarchical in the cross‐section, and the layers are called the oxide layer (region I), transition layer (region II) and matrix (region III). However, there is no hierarchical region in the TEM image of the cross‐sectional structure of the AN700 ribbon (Figure S5a,b, Supporting Information). The HRTEM image shows that region I mainly comprises nanocrystal structures of Fe_2_O_3_ and FeOOH (Figure 1c). Figure 1d,e shows the magnified Fe_2_O_3_ and FeOOH structures and corresponding FFT patterns, respectively. Second, the HRTEM image clearly shows that region II exhibits a unique heterogeneous structure, and the corresponding FFT indicates that the overall structure is amorphous (Figure 1f). Figure 1g shows the zoomed‐in selected region in Figure 1f, where two different amorphous regions can be distinguished: interfacial region II−1 and nanoscale grain region II−2. The corresponding FFT patterns for these two regions are shown in the upper right of Figure 1g, indicating that the two regions (interface and nanoscale grains) were amorphous, and had different diffraction halos, indicating differences in their compositions. Finally, the HRTEM image shows that numerous nanocrystal structures existed in the amorphous matrix in region III (Figure 1h), and the nanocrystal ring in the corresponding FFT pattern was Fe_3_P (Figure 1i). Notably, the microstructure of AN700 (region III) evolves into the fully amorphous structure (region II) again during the acid‐treated process, i.e., the heterogeneous amorphous structure with “Rejuvenation” characteristics.

Figure 1j,k, and Figure S6 (Supporting Information) show the high‐angle annular dark‐field scanning TEM (HAADF‐STEM) images and corresponding elemental distribution and content of the HG@AN700 ribbon. In region I, concentrated distribution of elements Fe and O is observed, and the ratio is ≈1:2 (Figure S6e, Supporting Information). Moreover, Fe, P, and O coexist in region II, element Fe as opposed to O. This demonstrates that the main elements in region II−1 are O and P, and the main elements in region II−2 are Fe and P. Meanwhile, the elements in region III are relatively homogeneously distributed, and element contents are close to the nominal composition, and Figure S5c (Supporting Information) also demonstrates the element homogeneously distributed of AN700 ribbon. Because the introduction of the C element is diversified, this study is only for reference data. Accordingly, the microstructure evolution during hierarchical gradient catalyst fabrication became clear. The results of structural characterizations revealed that the AS ribbon is a homogeneous FePC amorphous structure, and the AN700 ribbon is the coexistence of nanocrystal Fe_3_P and a homogeneous FePC amorphous structure. HG@AN700 ribbon is a hierarchical gradient catalyst that evolves to form nanocrystal FeOOH and “Rejuvenation” heterogeneous FeP/FePCO amorphous structure. Therefore, combined with the structural and chemical heterogeneities, it is speculated that the major active sites contributing to the degradation activity of dye solution would be homogeneous amorphous FePC (FePC), coexisting nanocrystal Fe_3_P and amorphous FePC (Fe_3_P/FePC), nanocrystal FeOOH (FeOOH), and “Rejuvenation” heterogeneous amorphous FeP/FePCO (FeP/FePCO), respectively (details in the following density functional theory (DFT) simulation).

### 2.2 Surface Analysis

**Figure 2:**
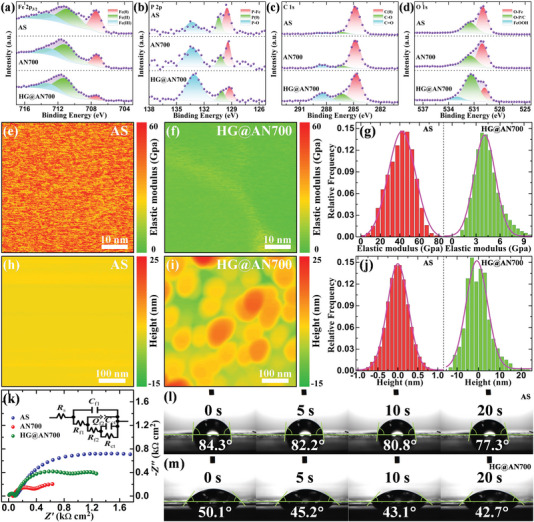
Surface analysis. XPS spectra of a) Fe 2p_3/2_, b) P 2p, c) C 1s, and d) O 1s of the original AS, AN700, and HG@AN700 ribbons. e,f) Maps of the surface elastic modulus on AS and HG@AN700 ribbons by AFM. g) Corresponding distributions of the surface elastic modulus of AS and HG@AN700 ribbons obtained from (e,f). h,i) Maps of the surface roughness (height) on AS and HG@AN700 ribbons by AFM. j) Corresponding distributions of the surface roughness (height) of AS and HG@AN700 ribbons obtained from (h,i). k) EIS Nyquist plots of original AS, AN700, and HG@AN700 ribbons in RR195 solution; the inset shows a general fitted circuit for EIS data. l,m) Contact angle test, the evolution of an RR195 solution water droplet on original AS and HG@AN700 ribbons surface at the initial stage.

To determine the discrepancy in the surface composition and electronic state evolution of the HG@AN700 ribbon during the fabrication process, we also performed X‐ray photoelectron spectroscopy (XPS) analysis for the elements Fe 2p_3/2_, P 2p, C 1s, and O 1s, as shown in **Figure** **2**a–d, and the XPS parameters are listed in Table S1. Figure 2a shows the XPS spectra of Fe 2p_3/2_ for the AS, AN700, and HG@AN700 ribbons. Three major peaks at approximately the binding energies of 707.0, 710.7, and 713.0 eV, corresponding to metallic Fe (Fe^0^), Fe^2+^, and Fe^3+^, respectively, are detected in the various ribbons. The fractions of the Fe^0^ peaks in the AS, AN700, and HG@AN700 ribbons were ≈31.47%, 20.09% and 9.06% of the total Fe 2p_3/2_ spectrum, respectively, and the fractions of the Fe^2+^ and Fe^3+^ peaks showed relative increases (Table S1, Supporting Information). These results suggest that the Fe^0^ on HG@AN700 gradually evolved into Fe^2+^ and Fe^3+^ during the fabrication process, forming Fe oxides and FeOOH, respectively. The P 2p XPS spectra of the various ribbons exhibited peaks at ≈129.4, 130.2, and 133.2 eV that were assigned to the compounds P–Fe, P^0^ and P–O, respectively (Figure 2b). The fraction of the P–O peak for HG@AN700 was higher than those for AS and AN700, owing to the chemical heterogeneity on the acid treatment ribbon surface. The C 1s XPS spectra consisted of three major peaks at ≈284.8, 286.4, and 288.4 eV, ascribed to C^0^, C─O and C═O, respectively (Figure 2c). The reason for the dramatic increase in the fraction of C─O + C═O peaks for the HG@AN700 ribbon surface was similar to that of P─O, both of which were owing to chemical heterogeneity caused by the acid treatment. The O 1s XPS spectrum shows two peaks that can be assigned to O─Fe and O─P/C, with binding energies of 530.0 and 531.5 eV, respectively, which were detected on the AS and AN700 ribbons. In comparison, three major peaks at around the binding energies of 529.8, 531.3, and 533.0 eV (FeOOH) were observed for the HG@AN700 ribbon (Figure 2d). Notably, in addition to the generation of the FeOOH species, the O−P/C peak also increased, indicating that FeOOH and O−P/C were more likely to exist in the acid treatment process in a water‐based environment, which is in good agreement with the TEM characterization in Figure 1. Moreover, the acid treatment process enhanced the atomic reconstruction of the HG@AN700 catalyst surface, resulting in structural and chemical heterogeneities on the surface. This may increase the concentration of FeOOH and O−P/C species and result in a robust electron hybridization phenomenon, thereby improving the subsequent catalytic degradation performance.^[^ ^11^, ^23^ ^]^

These characterizations prove that HG@AN700 has more significant structural and chemical heterogeneities than the other catalysts. However, catalytic performance depends more on the surface conditions of the catalyst. Recently, a cantilever resonance driven by an atomic force microscope (AFM) in frequency modulation mode has been used to express the local elastic modulus and energy state of “Rejuvenation” metallic glass.^[^ ^24^, ^25^ ^]^ Figure 2e,f exhibits the elastic modulus mapping of AS and HG@AN700, respectively, as measured by AFM. As shown in Figure 2g, HG@AN700 showed a lower and more heterogeneous distribution of elastic modulus compared to AS, which is consistent with previously reported results for its higher energy states.^[^ ^21^, ^26^ ^]^ And the overall elastic modulus distribution of AN700 was significantly lower than that of AS owing to its microstructural composition (Figure S7a,b, Supporting Information). Meanwhile, the DSC test results showed that the Δ*H*′ value of the heterogeneous amorphous structure in HG@AN700 is higher than that of the homogeneous amorphous structure in AS at every 100 nm thickness (Figure S8, Supporting Information), which also indicates that HG@AN7000 (region II) has a higher energy state than AS (Matrix). Figure 2h,i exhibits the surface roughness (height) mapping of AS and HG@AN700, respectively, as measured by AFM. Details of the height distribution of the surface morphology are shown in Figure 2j. The surface roughness of HG@AN700 was significantly higher than that of the AS, indicating an increase in the specific surface area. Catalysts with high specific surface areas are more likely to exhibit intrinsic activity, which further improves the overall catalytic efficiency of HG@AN700. The surface height details of AN700 are similar to those of AS (Figure S7c,d, Supporting Information) and agree with the SEM results (Figure S2, Supporting Information). Furthermore, the EIS plots shown in Figure 2k characterize the electrochemical reactions of the various catalysts in a dye solution. An equivalent circuit (R(C(R(Q(R(CR)))))) was used to fit the Nyquist plots (inset of Figure 2k), and the fitting results of the EIS plots are shown in Table S2 (Supporting Information). The Nyquist plot radius of HG@AN700 in the dye solution was smaller than that of AS and larger than that of AN700, indicating that the acid‐treatment process improved its corrosion resistance. Moreover, the fitting equivalent circuit model showed that HG@AN700 has a lower charge transfer resistance (*R*_ct_) ,^[^ ^27^ ^]^ which is conducive to the enhancement of charge transport and exerts a better catalytic effect in the dye degradation process.^[^ ^12^, ^21^ ^]^ The variation in the potential in the polarization curve is similar to that in the EIS results (Figure S9, Supporting Information). In addition, the contact angles were measured to study the hydrophilicity of the AS, AN700 and HG@AN700 ribbons (Figure 2l,m; Figure S10, Supporting Information). Notably, the contact angles of the water drops of HG@AN700 are much smaller than those of AS and AN700, and continue to decrease significantly in a short period (within 20 s). Because of the excellent hydrophilicity of HG@AN700, the surface of HG@AN700 had a larger contact surface area with the dye solution compared with AS and AN700 of the same size, which facilitated the contact between the dye molecules in the solution and active site, thus considerably improving the reaction rate.^[^ ^28^, ^29^ ^]^

### 2.3 Catalytic Performance

**Figure 3:**
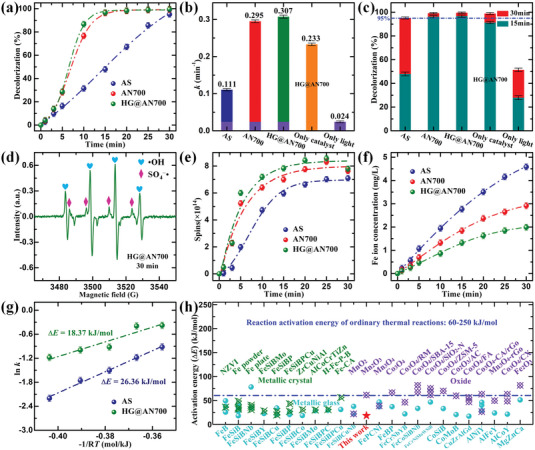
Catalytic performance. a) Comparison of RR195 solution degradations of original AS, AN700, and HG@AN700 ribbons. b) Catalytic degradation rate constant *k*. c) Decolorization efficiency. d) EPR spectrum of DMPO‐^•^OH/SO_4_^−•^. e) Total spin number (spins) of DMPO‐^•^OH/SO_4_^−•^ generated by original AS, AN700, and HG@AN700 ribbons in RR195 solution versus time. f) Fe ion concentration changes in RR195 solution. g) Arrhenius plots of ln(*k*) versus −1/R*T* for the calculation of the activation energy (Δ*E*). h) Comparison of activation energy (Δ*E*) for various metallic glass, metallic crystal, and oxide catalysts; further details are provided in Table S3 in the Supporting Information. The data are presented as the means values ± SEM (n = 3). The dash dot line is the experimental data fitting line.

**Figure** **3**a shows the catalytic degradation effect of the RR195 solution on the original AS, AN700, and HG@AN700 ribbons. HG@AN700 had a higher color removal ability than AS and AN700. Meanwhile, the two experimental control groups of “No light only HG@AN700” and “No catalyst only light” are also shown in Figure S11 (Supporting Information), indicating that “Only catalyst” and “Only light” can also decolorize the dye solution. Figure S12 (Supporting Information) summarizes the visible color changes of the RR195 solution over time under different experimental parameters. Typically, the reaction rate (*k*, which is fitted to the pseudo‐first‐order kinetic model, ln(*C*_0_/*C*_t_) = *k*t, where *C*_0_, the initial concentration (mg L^−1^), *C*_t_, the instant concentration at *t*, and decolorization efficiency (*η* = (1 − *C*_t_/*C*_0_) × 100%, *t* = 15 and 30 min) are the basic data that show the degradation ability of the catalyst toward the dye solution. As shown in Figure 3b, HG@AN700 shows the highest *k* (0.307 min^−1^), and “Only light” shows *k* = 0.024 min^−1^, indicating that the catalytic capacity of the ribbon itself plays a decisive role in the dye degradation process. Moreover, “Only light” can only degrade the dye solution through the decomposition of S_2_O_8_^2−^ into SO_4_^−•^ (S_2_O_8_^2−^ $\overset{hv}{\to }$ 2SO_4_^−•^).^[^ ^30^, ^31^ ^]^ Figure 3c shows the difference in decolorization efficiency between the different experimental parameters at 15 and 30 min.

To understand the types of reactive groups that contribute to the degradation reaction of the dye solution, DMPO was selected as the spin‐trapping reagent for the reactive species, and the electron paramagnetic resonance (EPR) spectra are shown in Figure 3d and Figure S13a–c (Supporting Information). The EPR spectrum showed both DMPO‐^•^OH and DMPO‐SO_4_^−•^ characteristic signal peaks (Figure 3d), indicating that the molecular structure of the dye was rapidly destroyed by both the generated hydroxyl radicals (^•^OH) and sulfate radicals (SO_4_^−•^). Accordingly, the chemical reactions during dye degradation include (Equations (1)−(3)):^[^ ^32^, ^33^ ^]^

(1) $${\mathrm{Fe}}^{0}+S_{2}O_{8}{{}^{2}}^{-}\to {\mathrm{Fe}}^{3+}+{\mathrm{SO}}_{4}^{-•}+{\mathrm{SO}}_{4}{{}^{2}}^{-}+2e^{-}$$

(2) $${\mathrm{SO}}_{4}^{-•}+H_{2}O\to {\mathrm{SO}}_{4}{{}^{2}}^{-}+{}^{•}\mathrm{OH}+H^{+}$$

(3) $${}^{•}\mathrm{OH}/{\mathrm{SO}}_{4}^{-•}+\mathrm{Organic}\to \mathrm{products}$$

Moreover, SO_4_^−•^ is more prone to electron transfer reactions than ^•^OH,^[^ ^34^ ^]^ in contrast to SO_4_^−•^, while ^•^OH is more likely to undergo hydrogen abstraction or addition.^[^ ^35^ ^]^ Thus, the intensity of the characteristic DMPO‐^•^OH signal peak in the EPR spectrum is higher than that of DMPO‐SO_4_^−•^ (Figure 3d), which also demonstrates that SO_4_^−•^ interconversion to ^•^OH can be rapidly induced in persulfate activation system.^[^ ^36^ ^]^ Meanwhile, in order to determine which of SO_4_^−•^ and ^•^OH plays a major role in the system. Based on the characteristics that ethanol (EtOH) and tertiary butanol (TBA) can quench SO_4_^−•^ and ^•^OH radicals, respectively,^[^ ^37^ ^]^ the quenching experiments were designed (Figure S13d, Supporting Information). The results show that adding EtOH (0.1 mol L^−1^) makes the decolorization rate lower than adding TBA (0.1 mol L^−1^), indicating that SO_4_^−•^ is the major reactive group in this system. Combined with the DMPO‐^•^OH/SO_4_^−•^ characteristic signal peak intensities in the EPR spectra of original AS, AN700, and HG@AN700 ribbons (Figure S13a–c, Supporting Information), the “Spins‐*t*” curves were constructed, as shown in Figure 3e. Notably, the total spin number of the radicals generated by HG@AN700 in the dye degradation process was higher than that of the other catalysts, which agrees with the decolorization process shown in Figure 3a. Furthermore, the concentration of Fe‐leached using HG@AN700 was much lower than that of the other catalysts during the catalytic degradation process (Figure 3f), and the contribution of the leached Fe ions was less than that of the catalyst itself, demonstrating that the various catalysts in the heterogeneous reaction primarily induced the dye degradation in this study.

The activation energy (Δ*E*) is an intrinsic kinetic characteristic that reflects the catalytic capacity of environmental catalysts and provides an important clue for explaining the amount of energy required to overcome the catalytic reaction barrier. The results of Δ*E* calculation, according to Arrhenius equation: ln*k* = −Δ*E*/*RT* + ln*A* (where *k* is the kinetic rate at different temperatures (*T*), *R* is the gas constant and *A* is a pre‐exponential factor), results indicate that HG@AN700 has a much lower Δ*E* than that of AS (Figure 3g; Figure S14, Supporting Information). As shown in Figure 3h and Table S3 (Supporting Information), the achieved Δ*E* value of metallic glass catalysts with various element components is generally lower than that of the metallic crystal and oxide catalysts, and the Δ*E* value of HG@AN700 catalyst in this study only is 18.37 kJ mol^−1^, demonstrating a much lower Δ*E* value than the ordinary thermal reactions (60−250 kJ mol^−1^) .^[^ ^38^ ^]^ From the perspective of potential energy,^[^ ^39^, ^40^ ^]^ compared to metallic crystals and oxide catalysts, metallic glasses with a nonequilibrium metastable nature are always in the top position of potential energy, and their Gibbs free energy is also relatively greater; thus, they have more potential energy to perform catalytic reactions.

### 2.4 Applicable Performance

**Figure 4:**
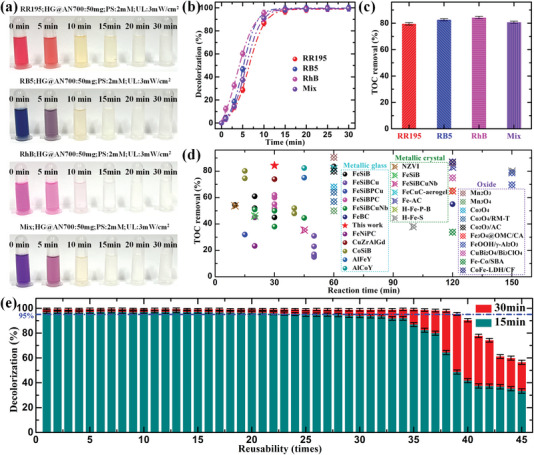
Applicable performance. a) Visible color change of various dyes solution. b) Decolorization efficiency of various dye solutions. c) TOC removal in various dyes solution using HG@AN700 ribbon catalyst. d) Comparison of TOC removal for various metallic glass, metallic crystal, and oxide catalysts; further details are provided in Table S4 in the Supporting Information. e) Reusability of the original HG@AN700 ribbon catalyst. The data are presented as the means values ± SEM (n = 3). The dash dot line is the experimental data fitting line.

**Figure** **4** shows the applicability performance of the HG@AN700 ribbons in the catalytic degradation of various dye solutions. As shown in Figure 4a, various dyes, including RR195, RB5, RhB, and mixed aqueous solutions with complex organic structures were gradually decolorized to colorlessness within 30 min using the HG@AN700 ribbon as the catalyst. The corresponding UV–vis absorbance spectra of the decolorization process for various dye solutions are shown in Figure S15 (Supporting Information). Notably, ≈100% of the color and ≈80% of the total organic carbon (TOC) were removed within 30 min for the four different dye solutions (Figure 4b,c), indicating that the HG@AN700 catalyst has a strong catalytic degradation effect for various dyes. The TOC removal rates achieved by the metallic glasses, metallic crystals and oxide catalysts at different times are summarized in Figure 4d and Table S4 (Supporting Information). Metallic glasses exhibited a higher TOC removal rate in a shorter period (within 60 min) than metallic crystals and oxides. By contrast, the metallic crystals and oxide catalysts exhibited a high TOC removal rate for a longer period (60−150 min). The TOC removal rate of the HG@AN700 catalyst in this study was 84.3% after 30 min, indicating that HG@AN700 can achieve high mineralization of the dye solution in a short time. Figure S16 (Supporting Information) shows the catalytic degradation of RR195 dye solution by HG@AN700 ribbon at different pH values and dye concentrations. The results show that with the increase of pH value (Original: pH = 5.4) and dye concentration, the dye decolorization rate will gradually decrease, but complete decolorization can still be achieved within 30 min.

The sustainability and stability of excellent catalysts are important indices for evaluating their application in environmental water remediation. Figure 4e and Figure S17 (Supporting Information) show the reusability of the AN700 and HG@AN700 ribbons for RR195 dye solution degradation. The reusability of the AS ribbon was not investigated because of its low *k* value. Surprisingly, compared to 24 times for AN700 ribbon, HG@AN700 ribbon can be reused ≈39 times while maintaining the nearly identical efficiency as the first use (*η* ≥ 95% at 30 min), followed by the “unyielding” decay process. The dye solution decolorization efficiency was sustained at ≈100% within 30 min using a 50 mg ribbon, 2 mM sodium persulfate, and little light irradiation. This highly efficient and durable environmental catalyst has significant potential for practical wastewater treatment applications. Moreover, the structural evolution and elemental changes in the ribbons during the reusability process are of considerable significance for analyzing catalyst characteristics. The XRD patterns of the reused AS, AN700, and HG@AN700 ribbons are shown in Figure S18 (Supporting Information). They retain their original structural characteristics. The SEM micrographs of the reused ribbons surface morphologies are shown in Figures S19−S21 (Supporting Information). In the reusability process, the original surface morphology of the catalysts will be gradually destroyed, and some residues formed in the catalytic degradation process will remain. The dry land shape structure on the reused ribbons surface after high cycle times may be associated with the decline of catalyst activity. The XPS spectra of the reused ribbons for Fe 2p_3/2_, P 2p, C 1s, O 1s, and S 2p are shown in Figures S22−S24 (Supporting Information), and the XPS parameters are listed in Table S1 (Supporting Information). In the reusability test, the zero‐valence Fe, P, and C on the HG@AN700 ribbon surface gradually changed to high‐valence states. The O−Fe peak fraction gradually decreased to zero, indicating the coexistence of O−P/C and FeOOH. Meanwhile, owing to the influence of sodium persulfate in the dye solution, a certain amount of sulfate ions were gradually deposited on the catalyst surface. The results for the reused AS and AN700 ribbons are similar to those for the reused HG@AN700 ribbons.

**Figure 5:**
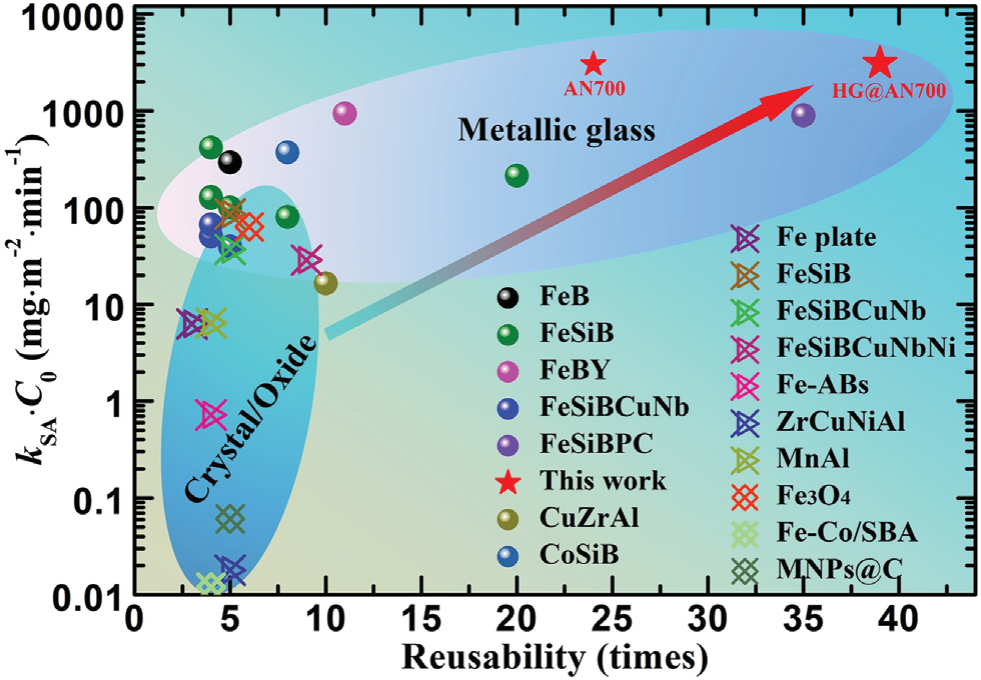
Comparison of catalytic performance. Degradation capability (*k*_SA_^•^ *C*_0_) versus reusability (times) for various metallic glass and crystal/oxide catalysts. An excellent catalyst should possess high degradation efficiency and strong reusability (top right‐hand area) (further details are provided in Table S5 in the Supporting Information).

To further highlight the excellent catalytic performance of the HG@AN700 catalyst, the degradation capability (*k*_SA_^•^ *C*_0_) versus reusability (times) for various metallic glass and crystal/oxide catalysts is summarized in **Figure** **5**, with more details are provided in Table S5. Fe ions have been used as standard industrial Fenton catalysts because of their highly reactive and homogeneous nature.^[^ ^41^, ^42^ ^]^ However, defects in ion‐state catalysts, such as one‐time usability and secondary pollution by metallic sludge, have become major factors hindering their industrial application.^[^ ^43^ ^]^ By contrast, metallic crystals and oxide catalysts have the advantages of low cost, easy fabrication, and high efficiency and are known as iterative Fenton catalysts. However, because of the influence of microstructural defects (poor corrosion resistance, easy surface decay, and high resistivity caused by grain boundaries), the catalytic degradation ability and reusability of metallic crystals and oxide catalysts are limited.^[^ ^44^ ^]^ As shown in Figure 5, crystal/oxide catalysts are reusable up to 10 times. Compared to crystal/oxide alternatives, metallic glass catalysts exhibit high degradation ability and strong reusability when degrading dye solutions. Surprisingly, the HG@AN700 ribbon in this study demonstrated the best performance, with both ultrahigh degradation ability of *k*_SA_^•^ *C*_0_ = 3101 mg m^−2^ min^−1^ and ultrastrong reusability of 39 times without efficiency decay. This compelling design mechanism for hierarchical‐gradient Fe‐based metallic glass catalysts may provide a direction for new, undeveloped catalysts with higher performance.

### 2.5 DFT Simulation

**Figure 6:**
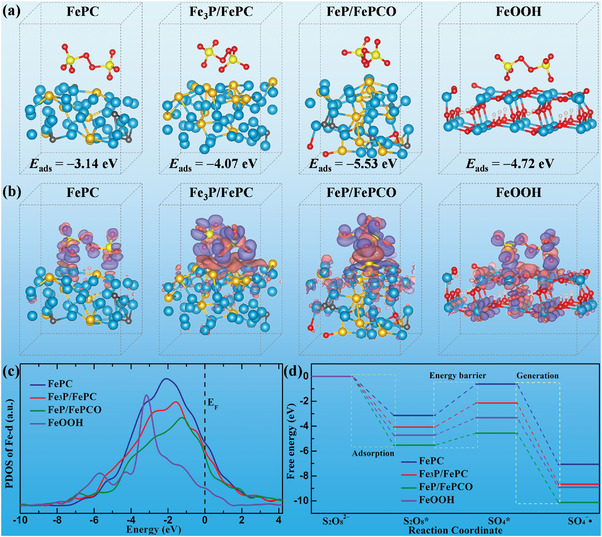
DFT simulation. a) DFT calculation *E*_ads_ of S_2_O_8_^2−^ on the surfaces of the FePC, Fe_3_P/FePC, FeP/FePCO, and FeOOH atomic configurations. b) Corresponding electron density differences of these configurations are also shown: pink and violet isosurfaces represent the depletion and accumulation of electrons, respectively. c) Partial density of states (PDOS) of *d* orbital of Fe atom on the surfaces with adsorbed S_2_O_8_^2−^; the black dashed lines at zero energy indicate the Fermi level. d) Theoretical calculation free energy diagrams for S_2_O_8_^2−^ to SO_4_^−•^ conversion on the surfaces.

To reveal the origin of the excellent dye catalytic degradation activity, density functional theory (DFT) calculations were conducted to achieve an atomic‐scale understanding of various metallic glass catalysts.^[^ ^45^ ^]^ In particular, four representative atomistic models, FePC, Fe_3_P/FePC, FeP/FePCO, and FeOOH, were constructed to investigate their catalytic performance. According to the aforementioned experimental results, dye degradation involves a series of chemical reactions occurring at the solid−liquid interface and the initial step is the adsorption of S_2_O_8_^2−^ on the catalyst surface.^[^ ^12^ ^]^ Accordingly, the Δ*E*_ads_ of the surfaces of the various catalyst models were calculated, as shown in **Figure** **6**a. The results revealed that the FeP/FePCO exhibited the highest Δ*E*_ads_ value of −5.53 eV; in comparison, the Δ*E*_ads_ values of FePC, Fe_3_P/FePC, and FeOOH were −3.14, −4.07, and −4.72 eV, respectively. Surprisingly, FeOOH exhibited higher adsorption energy than FePC and Fe_3_P/FePC. To further explain the principle of enhanced S_2_O_8_^2−^ adsorption, Figure 6b presents the DFT simulations of the corresponding electron density difference after S_2_O_8_^2−^ adsorption onto the four models. Typically, the interaction between FeP/FePCO and S_2_O_8_^2−^ exhibited the strongest charge transferability, followed by that between Fe_3_P/FePC and S_2_O_8_^2−^, the interaction between FePC and S_2_O_8_^2−^ showed the weakest charge transferability. Moreover, the interaction between FeOOH and S_2_O_8_^2−^ showed strong charge transferability. The sequence of charge transferability is consistent with the adsorption energy, demonstrating stronger charge transferability and a higher Δ*E*_ads_ value.

To further elucidate the outstanding catalytic degradation performance of the dye, the partial density of states (PDOS) of the four atomistic models after S_2_O_8_^2−^ adsorption was calculated (Figure 6c; Figure S25, Supporting Information). As shown in Figure 6c, the FeOOH PDOS of Fe‐d shows a noticeable spike peak compared to the others, indicating that the electronic localization state is highly concentrated, which may be related to its unique crystal electronic structure.^[^ ^11^ ^]^ Meanwhile, the simulated results for FePC, Fe_3_P/FePC, and FeP/FePCO show that the central peaks of the PDOS of Fe‐d successively shift closer to the Fermi level (E_F_), and the proximity of the highest occupied d‐band electron to the Fermi level facilitates electron donation during adsorption, contributing to the adsorption of S_2_O_8_^2−^.^[^ ^46^, ^47^, ^48^ ^]^ Moreover, the electronic interaction between Fe‐d and O‐p for FeP/FePCO and FeOOH was much stronger than that of the other atomic orbitals (Figure S25, Supporting Information). Combined with their Δ*E*_ads_ values and the charge alteration around the oxygen atom in the electron density difference results, this indicates that the oxygen atoms play an active role in the adsorption of S_2_O_8_^2−^. With regard to the reaction pathway activity of S_2_O_8_^2−^ → S_2_O_8_∗ → SO_4_∗ → SO_4_^−•^,^[^ ^49^ ^]^ relaxed configurations of the four models and their corresponding intermediate structures are shown in Figure S26 (Supporting Information). Figure 6d shows the free energy profiles of the S_2_O_8_^2−^ to SO_4_^−•^ conversion process for the four models. First, the S_2_O_8_^2−^ → S_2_O_8_∗ adsorption energies at the FeP/FePCO, FeOOH, Fe_3_P/FePC, and FePC surfaces successively decreased. Second, notably, S_2_O_8_∗ dissociation on the surfaces of FePC, Fe_3_P/FePC, FeP/FePCO, and FeOOH is a nonspontaneous process. The energy barrier on the FeP/FePCO (0.97 eV) surface was much lower than those on the FeOOH (1.40 eV), Fe_3_P/FePC (1.93 eV), and FePC (2.52 eV) surfaces, and the lowest energy barrier for the FeP/FePCO surface indicated that it had the highest ability to cleave the O−O bond.^[^ ^49^ ^]^ Compared with other catalyst surfaces, the energy barriers of the rate‐determining steps (RDS) for SO_4_^−•^ generation on the FeP/FePCO surface were the lowest, suggesting easier stabilization of the SO_4_∗ intermediates.^[^ ^50^ ^]^ Finally, FeP/FePCO exhibited the smallest generation energy (−5.56 eV), whereas, the generation energies of FeOOH, Fe_3_P/FePC, and FePC were −5.59, −6.52, and −6.44 eV, respectively. The aforementioned theoretical calculations demonstrate that the interaction of the heterogeneous nanoglass structure results in a high adsorption energy, low energy barrier, and low generation energy, thus facilitating S_2_O_8_^2−^ to SO_4_^−•^ conversion, which is consistent with the experimental results.

## 3 Conclusion

In summary, we successfully fabricated a Fe‐based hierarchical gradient catalyst with a nanoscale chemically heterogeneous glassy structure by introducing oxygen atoms using non‐noble metals. The catalyst exhibited excellent performance, such as high rate, strong TOC removal, and low activation energy in degrading dye wastewater. Compared with the original Fe_75_P_15_C_10_ catalyst, the catalytic ability (*k*_SA_^•^ *C*_0_) of the hierarchical gradient catalyst increased from 1144 to 3101 mg m^−2^ min^−1^. Surprisingly, in stability testing, the developed catalyst could be continuously reused 39 times without significant efficiency decay, which is one of the best catalytic degradation ability and stability of environmental catalysts reported to date. Theoretical calculations confirmed that the unique heterogeneous nanoglass structure in the hierarchical gradient catalyst has strong charge transferability, resulting in a high adsorption energy, low energy barrier, and low generation energy in the process of facilitating S_2_O_8_^2−^ to SO_4_^−•^ conversion. The infiltration of O and the formation of FeOOH nanocrystals in the hierarchical gradient catalyst also played a positive role in the catalytic degradation of dye wastewater. This work provides a universal and facile method for fabricating nanostructured metallic glass and reveals that its chemically heterogeneous glassy structure is a new strategy for obtaining high catalytic degradation performance. Importantly, it provides a reliable research direction for the design of efficient and low‐cost environmental catalysts.

## Conflict of Interest

The authors declare no conflict of interest.

## Supporting information

Supporting Information

## Data Availability

The data that support the findings of this study are available from the corresponding author upon reasonable request.
