# Supplementary material for: Nanoscale Oxygenous Heterogeneity in FePC Glass for Highly Efficient and Reusable Catalytic Performance
Source: Adv Sci (Weinh). 2023 Sep 21;10(31):2304045. doi: 10.1002/advs.202304045 (PMC10625099; doi:10.1002/advs.202304045)
Supplement: Supplementary file 1 — Supporting Information [file ADVS-10-2304045-s001.pdf]

ADVANCED SCIENCE
Open Access

Supporting Information

for *Adv. Sci.*, DOI 10.1002/advs.202304045

Nanoscale Oxygenous Heterogeneity in FePC Glass for Highly Efficient and Reusable Catalytic Performance

*Qi Chen, Lingyu Guo, Haoxiang Di, Zhigang Qi, Zhaoxuan Wang, Ziqi Song, Laichang Zhang, Lina Hu and Weimin Wang**

Supporting Information

Nanoscale Oxygenous Heterogeneity in FePC Glass for Highly Efficient and Reusable Catalytic Performance

Qi Chen, Lingyu Guo, Haoxiang Di, Zhigang Qi, Zhaoxuan Wang, Ziqi Song, Laichang Zhang, Lina Hu, and Weimin Wang*

Q. Chen, Z. Qi, Z. Wang, Z. Song, L. Hu, W. Wang

Key Laboratory for Liquid-Solid Structural Evolution and Processing of Materials (Ministry of Education)

School of Materials Science and Engineering

Shandong University

Jinan 250061, China

E-mail: weiminw@sdu.edu.cn

L. Guo

School of Transportation and Logistics Engineering

Wuhan University of Technology

Wuhan 430063, China

H. Di

School of Chemical Engineering and Light Industry

Guangdong University of Technology

Guangzhou 510006, China

L. Zhang

School of Engineering

Edith Cowan University

270 Joondalup Drive, Joondalup, Perth, WA6027, Australia

Contents

1. Experimental

1.1. Materials

The raw metals, including high-pure Fe (99.99% wt.%), C (99.99% wt.%) and pre-alloyed Fe-P ingot (consisting of 71.5% at.% Fe and 28.5 at.% P) were purchased form Beijing Jiaming Platinum Nonferrous Metals Co., Ltd and mixed to atomic compositions of $Fe_{75}P_{15}C_{10}$ for metallic glass preparation. Commercially available reactive red 195 (RR195, $C_{31}H_{19}ClN_7Na_5O_{19}S_6$, AR grade), reactive black 5 (RB5, $C_{26}H_{21}N_5Na_4O_{19}S_6$, AR grade) and rhodamine B (RhB, $C_{28}H_{31}ClN_2O_3$, AR grade) were purchased from Shanghai Macklin Biochemical Co., Ltd. Sodium persulfate (PS, $Na_2S_2O_8$, AR grade) was purchased from Shanghai Macklin Biochemical Co., Ltd. Nitric acid (HNO_3, AR grade), Hydrochloric acid (HCl, AR grade), Sodium hydroxide (NaOH, AR grade), ethanol (EtOH) and tertiary butanol (TBA) were purchased from Sinopharm Chemical Reagent Co., Ltd. Milli-Q water (18.25 MΩ cm) was employed throughout this work for chemical dilution.

1.2. Materials Fabrication

Metallic glass ribbons with the atomic compositions of $Fe_{75}P_{15}C_{10}$ were prepared by our previously reported melt-spinning technique. Typically, master alloy ingots (5g) were initially fabricated by mixing and melting high-purity metals an arc-melting technique under an argon (Ar) atmosphere protection. To ensure the elements formed a homogeneous distribution, the master alloys were re-melted at least five times. Afterward, the master alloy ingots were remelted in quartz tube by inducting melting,

followed by single-roller melt spinning system in a purified argon atmosphere (roller tangent speed = 36 m s^{-1}) to obtain as-spun $Fe_{75}P_{15}C_{10}$ metallic glass ribbon (AS ribbon). The AS ribbon were annealed at 700 K for 300 min in a vacuum chamber under an argon (Ar) atmosphere protection followed by furnace cooling to obtain the AN700 ribbon. With respect to the acid-immersing process, the AN700 ribbon was immersed in 0.001 mol/L HNO_3 solution for 120 min at temperature of 323 K, then cleaned and dried to obtain HG@AN700 ribbon. The treated and used ribbons were preserved in an absolute ethanol solution for the subsequent experiments and characterizations.

1.3. Materials Characterization

The X-ray diffraction (XRD, Bruker D8 Discover) with Cu-Kα radiation, high resolution transmission electron microscope (TEM, FEI Talos F200), scanning electron microscope (SEM, JSM-7800F) equipped with an energy dispersive X-ray spectrometer (EDS), and atomic force microscope (AFM, Bruker Bioscope Resolve) were used for the characterization of the structural analysis and morphology of the ribbons. The high-angle annular dark-field scanning TEM (HAADF-STEM) images were taken with a FEI Talos F200 electron microscope with a highly sensitive energy dispersive X-ray spectroscopy detector system. Catalyst surface chemical compositions and valence states were characterized by X-ray photoelectron spectroscopy (XPS, AXIS Supra) with a monochromatic Al Kα X-ray source. The differential scanning calorimetry (DSC, Netzsch-404) under a purified Ar flow were used to determine the amorphous structure. The polarization curves and

electrochemical impedance spectroscopy measurements were carried out on an electrochemical measuring instrument (CHI 660E), and in the RR195 dye solution. The specific surface area (SSA) of the ribbons was measured by surface area and porosimetry analyzer (V-Sorb 2800P), and AS/AN700 = 0.0193 m^2/g, HG@AN700 = 0.0199 m^2/g. The contact angle (CA) of the ribbons was measured by optical contact angle measuring instrument (SDC-100). The pH-meter (PHS-3E) was used to determine the pH value of solution.

1.4. Catalytic Properties

The catalytic performance measurements using the as-prepared ribbons were carried out for RR195, RB5, RhB, and Mix dyes degradation. If not specifically mentioned, each catalytic experiment involved a dye concentration of 100 mg L^{-1}, catalyst dosage of 50 mg in 100 mL dye solution, and PS concentration of 2 mM, and the Mix dye is the sum of three equal parts of RR195, RB5 and RhB dyes. The ultraviolet irradiation (3 mW/cm^2) required for the catalytic degradation test was obtained by combining the filter with the simulated sunlight xenon lamp light source (PL-X500), and the dye solution was stirred at a fixed speed (200 r min^{-1}) during the catalytic degradation process. The target dye solution assessments were conducted in a thermostatic water bath to adjust the constant temperature (i.e., 298, 308, 318, 328 and 338 K). The dye solution (3 mL) were taken out at various time intervals of 0, 1, 3, 5, 10, 15, 20, 25 and 30 min followed by determination of UV-Vis spectrophotometer (MAPADA P9) to obtain the absorbance spectrum for dye decolorization. For reuse experiments, each reused ribbon was washed with Milli-Q

water three times followed by preservation in absolute ethanol solution. The total organic carbon (TOC) of the various dyes solution was determined with a TOC-L CPH analyzer. The inductively coupled plasma-mass spectrometry (ICP-MS, ICAP7400) was used to determine the concentration of iron ion in the various dyes solution. The electron paramagnetic resonance spectrometer (EPR, Bruker EMXPLUS) was carried out to identify the existence of certain reactive species and 5,5-dimethyl-1-pyrroline N-oxide (DMPO) was selected as a spin-trapping reagent for reactive species.

1.5. DFT Calculation Method

The ab initio molecular dynamics (AIMD) simulations were performed with the VASP code to produce various amorphous models. The module was first melt and equilibrated at a high temperature of 2000 K for 10 ps at constant volume with a Nosé thermostat (~1000 K above the melting temperature), Then quenched down to 300 K by velocity scaling over 2 ps, and well-relaxed for 5 ps at 300 K to obtain sufficient configurations. A 2 × 2 × 2 k-point grid was used to keep the computational cost at a reasonable level, and the MD time step was set to 1 fs. Four representative atomistic models, including FePC, Fe_3P/FePC, FeP/FePCO and FeOOH were constructed based on our experimental characterization to investigate their overall catalytic behaviors. Spin-polarized first-principle calculation was performed in the framework of density functional theory as implemented in the VASP program [1]. The generalized gradient approximation method with the Perdew-Burke-Enzerh function (GGA-PBE) was employed for the electronic exchange and correlation. The plane wave

pseudopotential with a kinetic cutoff energy of 450 eV within the projector augmented wave (PAW) method was used [2, 3]. The self-consistent the total energy convergence criteria were less than 10^{-5} eV and the geometry optimization were terminated when the forces on all atoms were smaller than 0.03 eV $Å^{-1}$. The k-point was generated by the Monkhorst-Pack grid method with 3 × 3 × 1 for geometry optimization [4]. The Van der Waals dispersion-corrected DFT (DFT–D3) was also carried out, as proposed by Grimme et al [5]. For all models the vacuum space along the z-direction was set to be 15 Å, which was enough to avoid interaction between the two neighboring images. The barriers for $S_2O_8^{2-}$ decomposition are calculated with the climbing-image nudged elastic band (CI-NEB) method [6].

1.6. Statistical Analysis

Pre-processing of data was performed with either CasaXPS, ZRxModel, Excel and DigitalMicrograph package. All data were expressed as mean ± SEM (standard error of mean). Unless otherwise mentioned, n = 3, i.e. each experiment was tested three times in parallel. Statistical analysis of the data was performed using "Descriptive Statistics" in Origin software, and the data points were fitted using Gauss and Boltzmann.

2. The Supplementary Figures and Tables

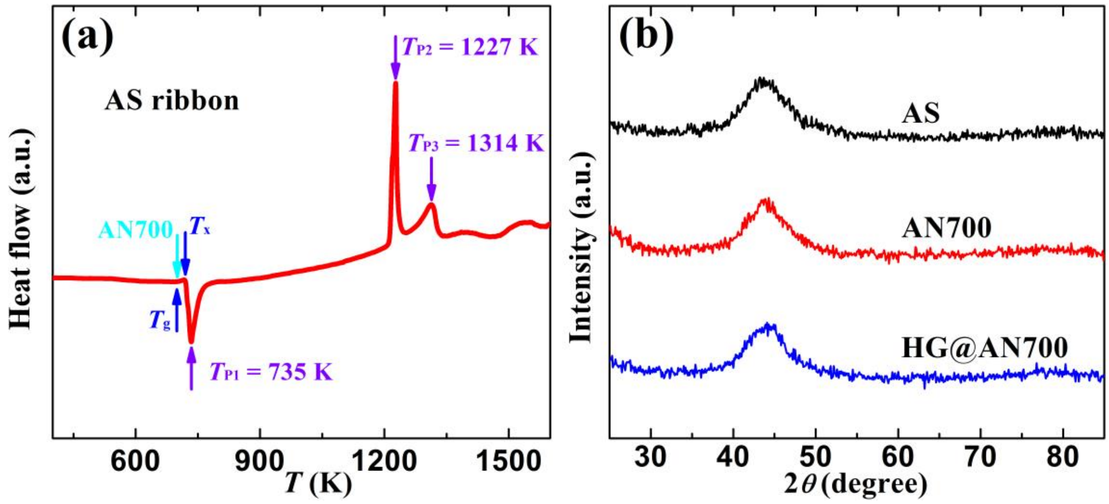

Figure S1. a) DSC curve of original AS ribbon. b) XRD patterns of original AS, AN700, and HG@AN700 ribbons.

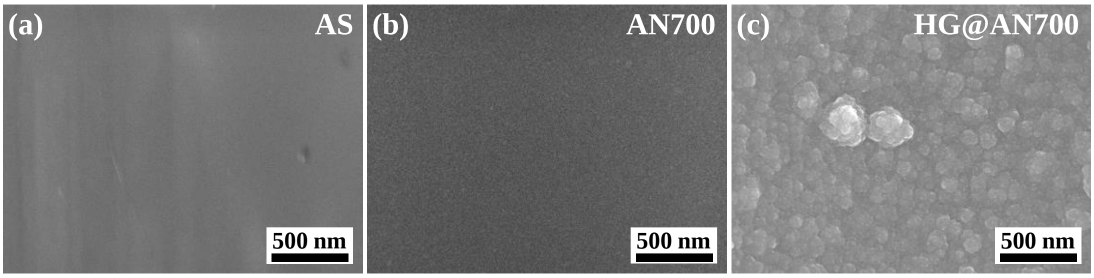

Figure S2. a-c) SEM micrographs of surface morphologies of original AS, AN700 and HG@AN700 ribbons, respectively.

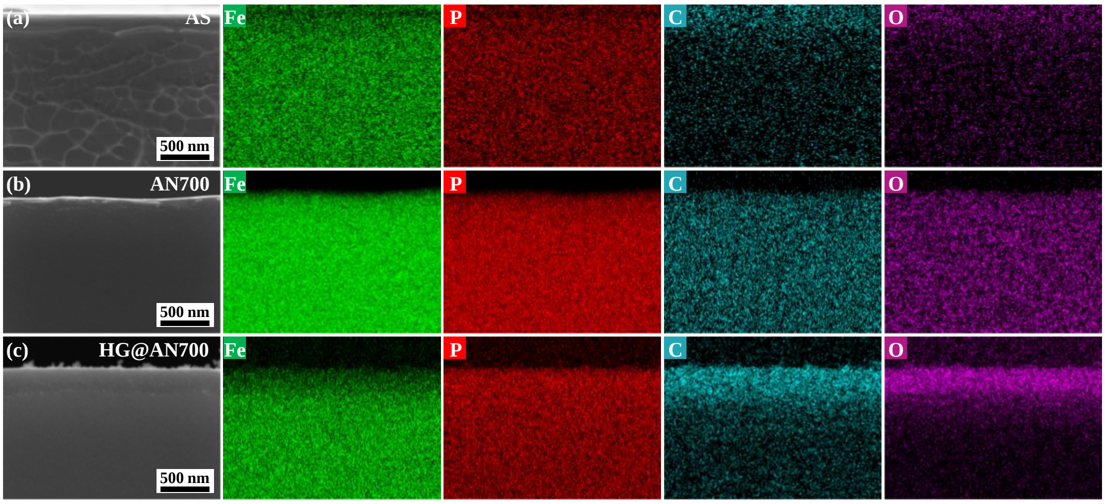

Figure S3. a-c) SEM micrographs of cross-sectional structure of original AS, AN700 and HG@AN700 ribbons, respectively; and elemental mapping results of Fe, P, C and O.

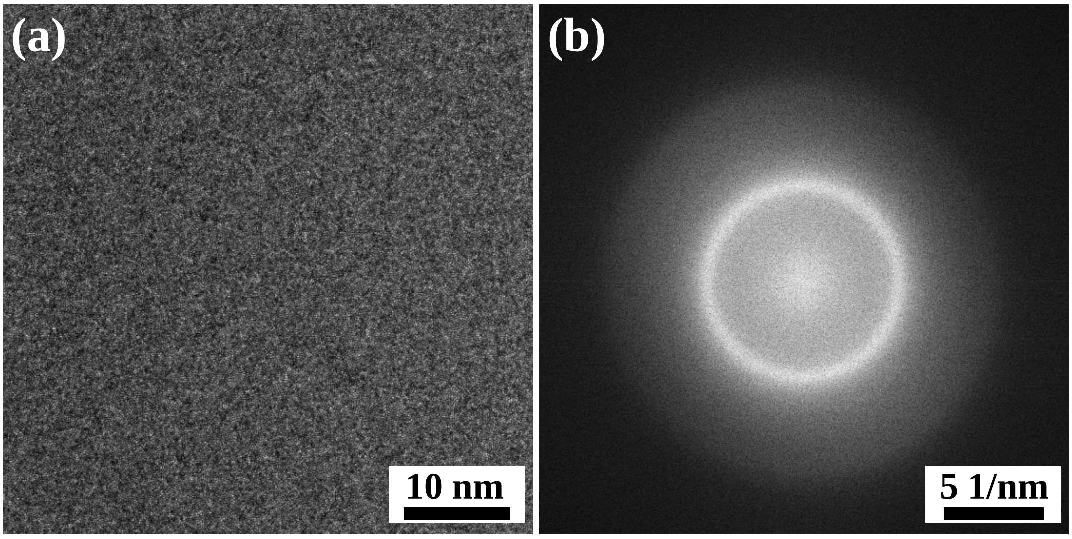

Figure S4. TEM characterizations of original AS ribbon; a) HRTEM image and b) FFT pattern of original AS ribbon.

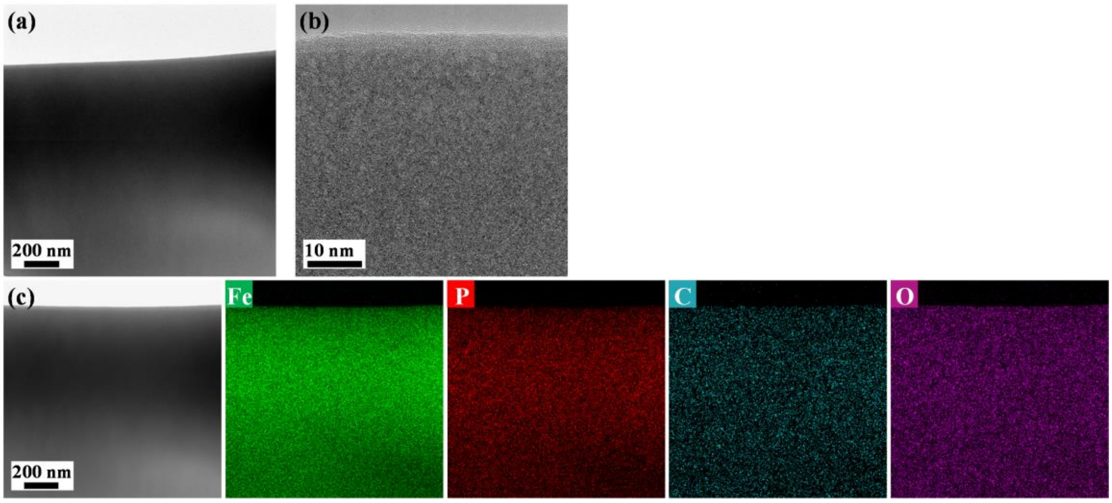

Figure S5. a–b) TEM image of the cross-sectional structure of AN700 ribbon. c) HAADF-STEM images of the cross-sectional structure of AN700 ribbon.

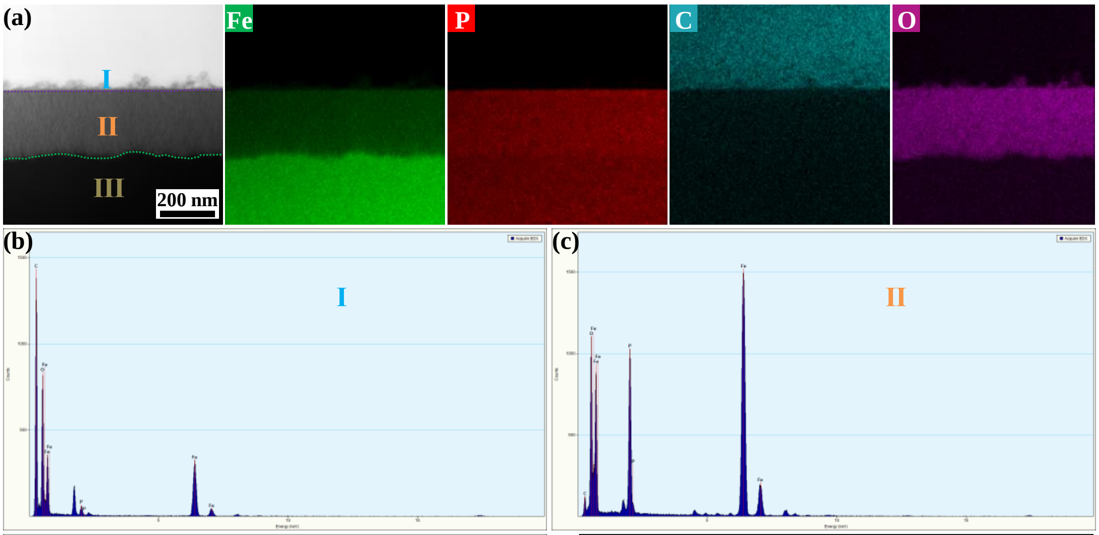

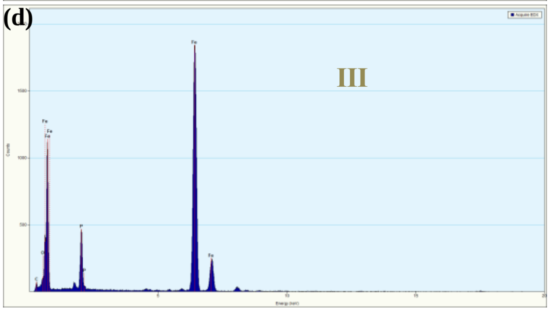

(e)

Region	Atomic ratio (%)			
	c_{Fe}	c_P	c_C	c_O
I	10.35	0.52	69.73	19.40
II	46.07	17.12	11.36	25.45
III	72.15	15.85	10.48	1.52

Figure S6. a) HAADF-STEM images of the cross-sectional structure of original HG@AN700 ribbons; and elemental mapping results of Fe, P, C and O. b-d) EDX patterns for the three regions in (a). e) Corresponding EDX data results.

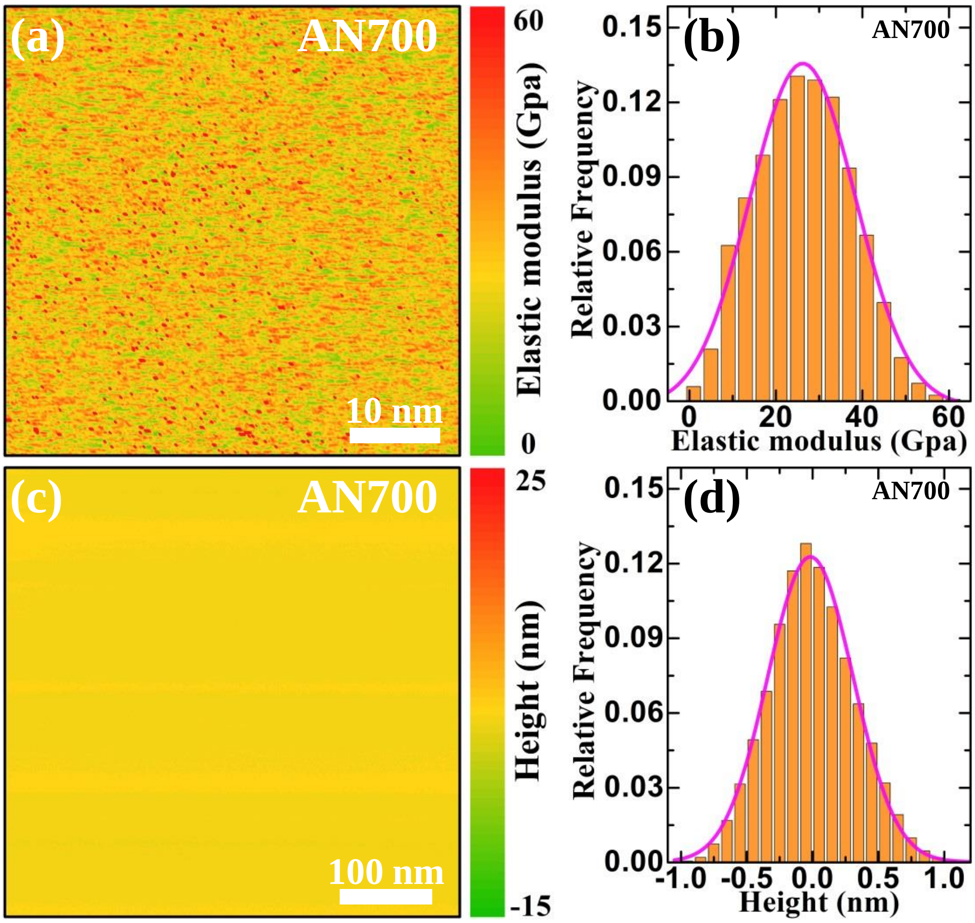

Figure S7. a) Map of the surface elastic modulus on AN700 ribbon by AFM. b) The corresponding distributions of the surface elastic modulus of AN700 ribbon obtained from (a). c) Map of the surface roughness (height) on AN700 ribbon by AFM. d) The corresponding distributions of the surface roughness(height) of AN700 ribbon obtained from (c).

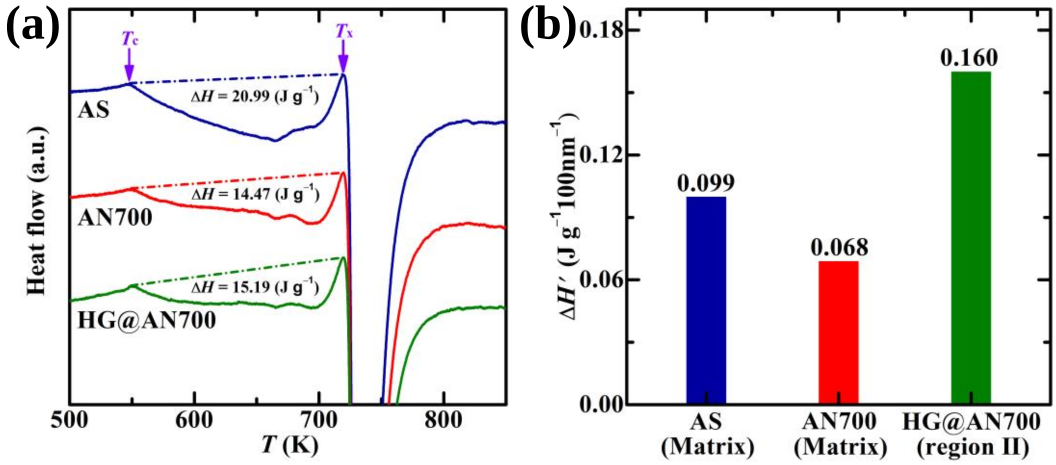

Figure S8. a) The DSC curves of AS, AN700 and HG@AN700 ribbons with baselines subtracted focusing on the temperature range of T_c–T_x showing the ΔH. b) The $\Delta H'$ values of every 100 nm thickness of AS (Matrix), AN700 (Matrix) and HG@AN700 (region II) ribbons.

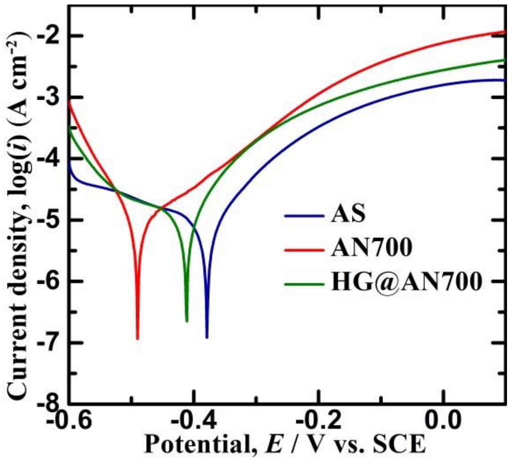

Figure S9. The polarization curves of original AS, AN700 and HG@AN700 ribbons in RR195 solution.

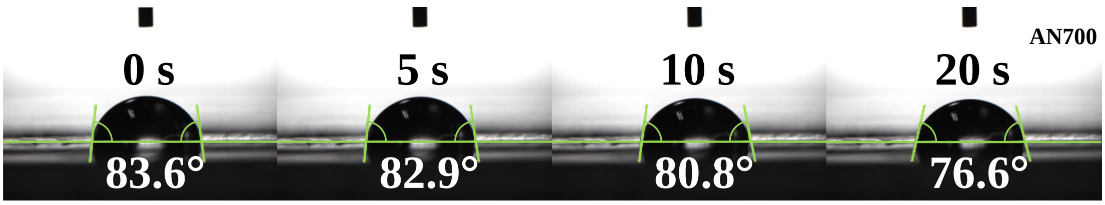

Figure S10. Contact angle test, the evolution of a RR195 solution water droplet on original AN700 ribbon surface at the initial stage.

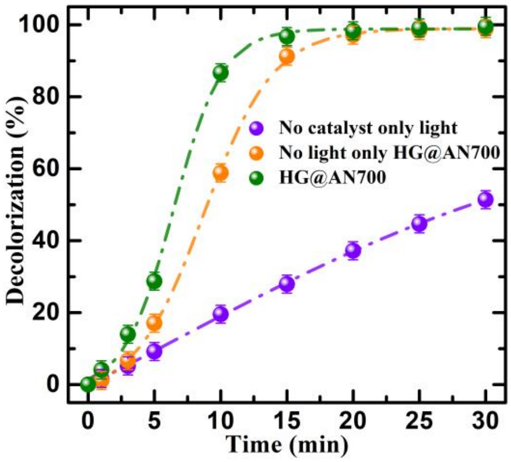

Figure S11. Comparison of RR195 solution degradations under different conditions. The data are presented as the means values ± SEM (n = 3). The dash dot line is the experimental data fitting line.

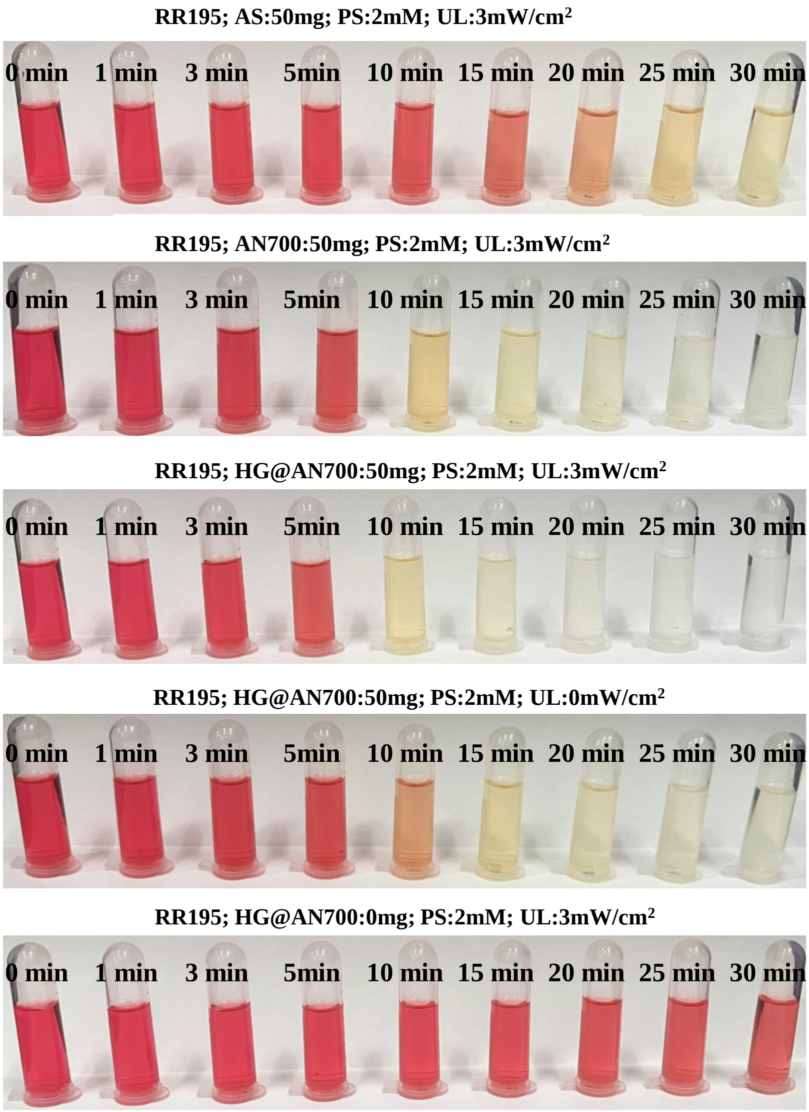

Figure S12. Visible color change of RR195 solution under different experimental parameters.

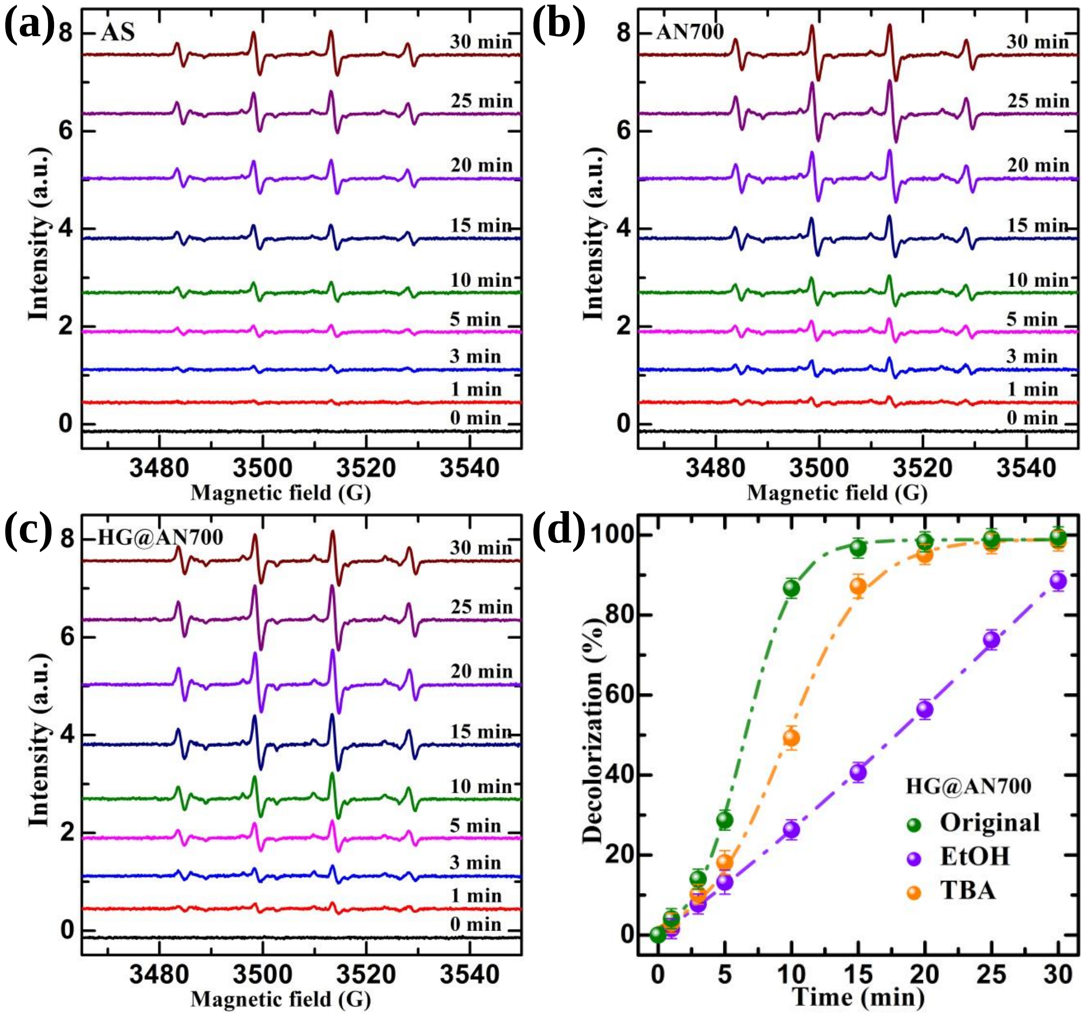

Figure S13. a-c) EPR spectra of DMPO-•OH/SO_4^{-}• generated by original AS, AN700 and HG@AN700 ribbons in RR195 solution, respectively. d) Comparable results of decolorization efficiency of HG@AN700 ribbon with and without adding quenching agents of EtOH and TBA in RR195 solution. The data are presented as the means values ± SEM (n = 3). The dash dot line is the experimental data fitting line.

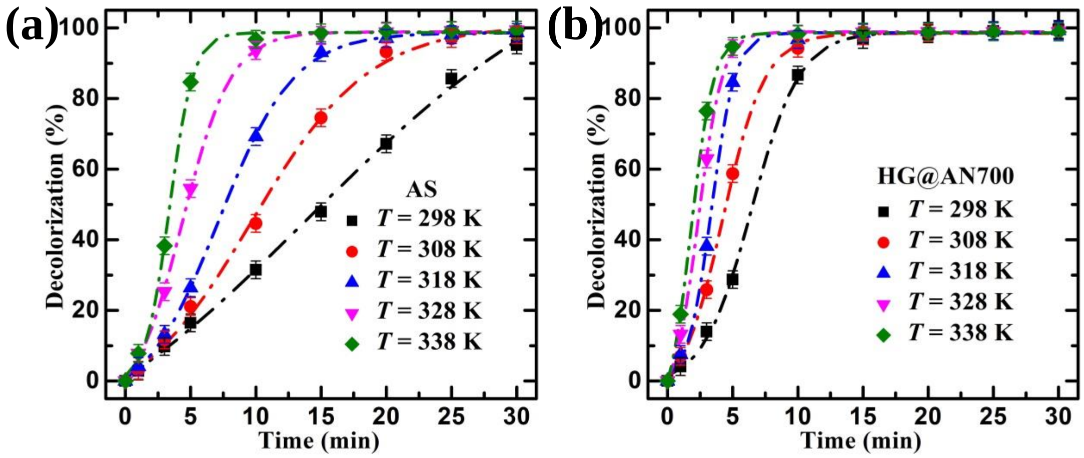

Figure S14. Effects of reaction temperature on dye degradation using original a) AS and b) HG@AN700 ribbons. The data are presented as the means values ± SEM (n = 3). The dash dot line is the experimental data fitting line.

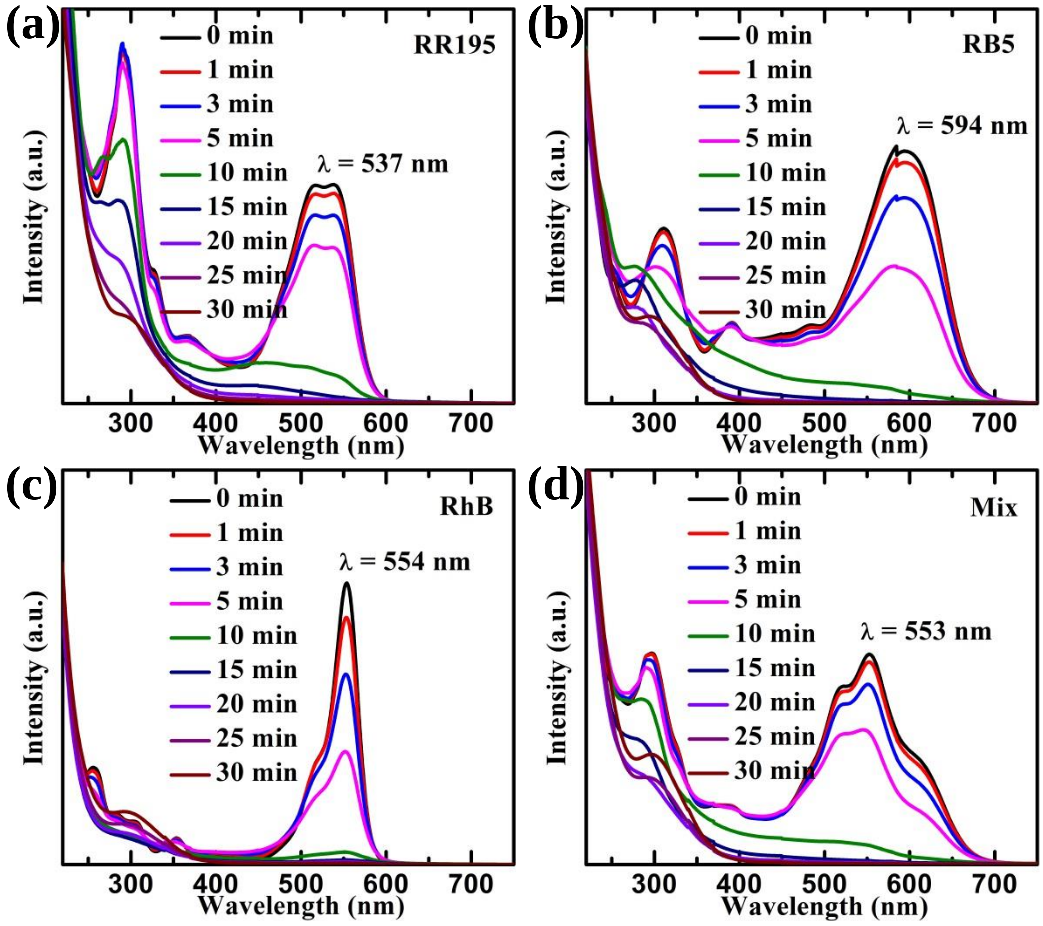

Figure S15. UV-Vis absorbance spectra of a) RR195, b) RB5, c) RhB and d) Mix dyes solution at different time intervals using original HG@AN700 ribbon.

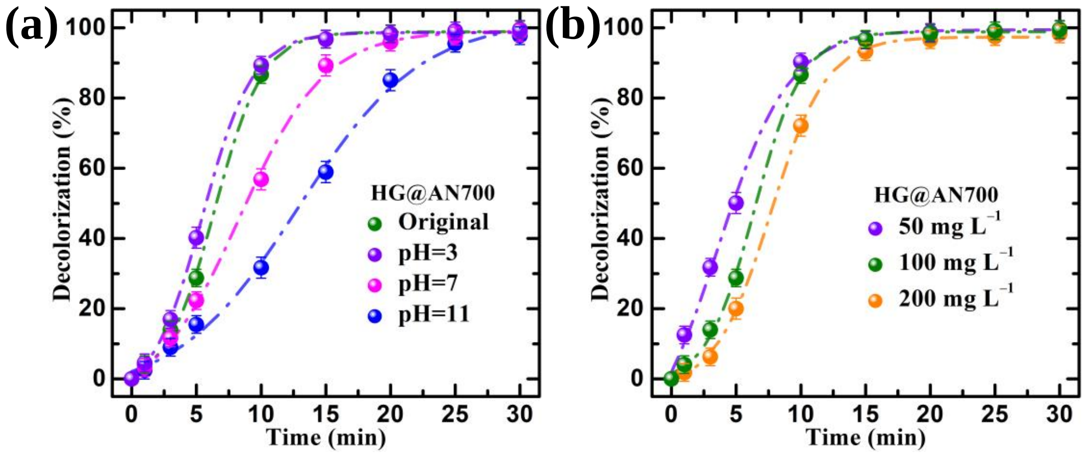

Figure S16. a) The decolorization efficiency of RR195 dye solution of HG@AN700 ribbon at different pH values. b) The decolorization efficiency of RR195 dye solution of HG@AN700 ribbon at different dye concentrations. The data are presented as the means values ± SEM (n = 3). The dash dot line is the experimental data fitting line.

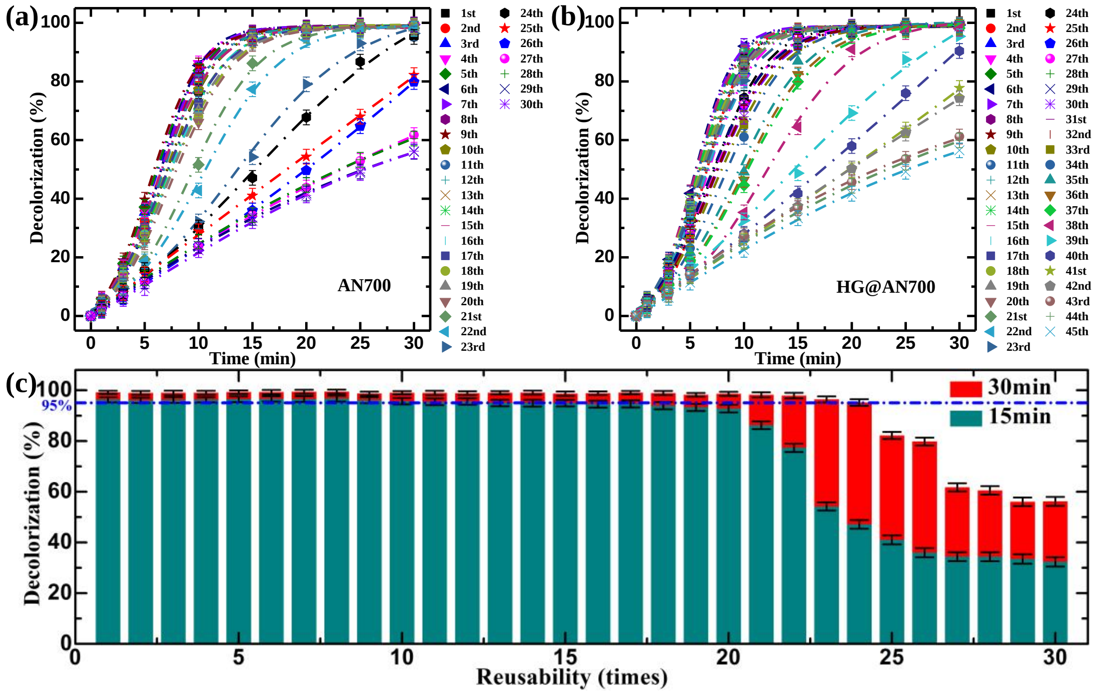

Figure S17. Decolorization efficiency of RR195 solution with *t* during the catalytic degradation process by original a) AN700 and b) HG@AN700 ribbons from the different degradation cycles. c) Reusability of the original AN700 ribbon catalyst. The data are presented as the means values ± SEM (n = 3). The dash dot line is the experimental data fitting line.

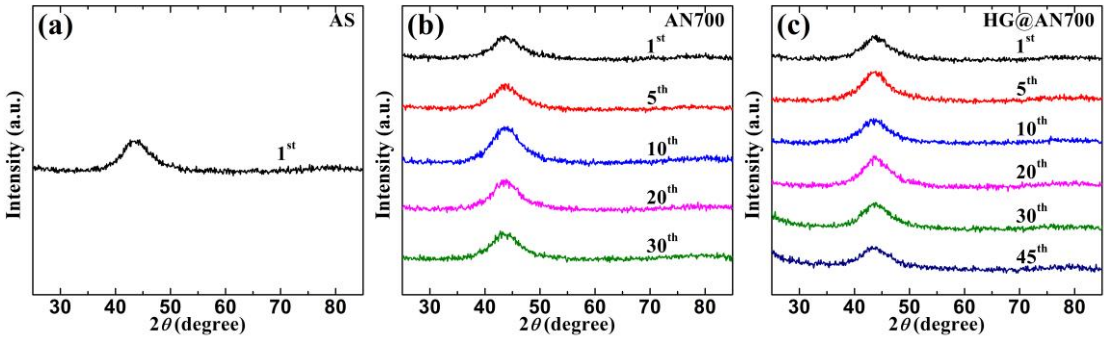

Figure S18. a-c) XRD patterns of the reused AS, AN700 and HG@AN700 ribbons respectively.

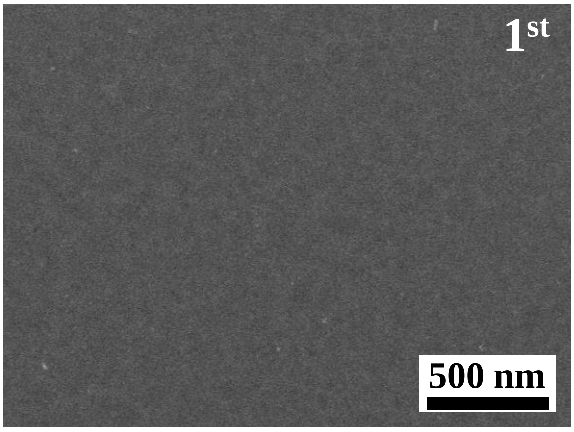

Figure S19. SEM micrographs of surface morphologies of the reused AS ribbon.

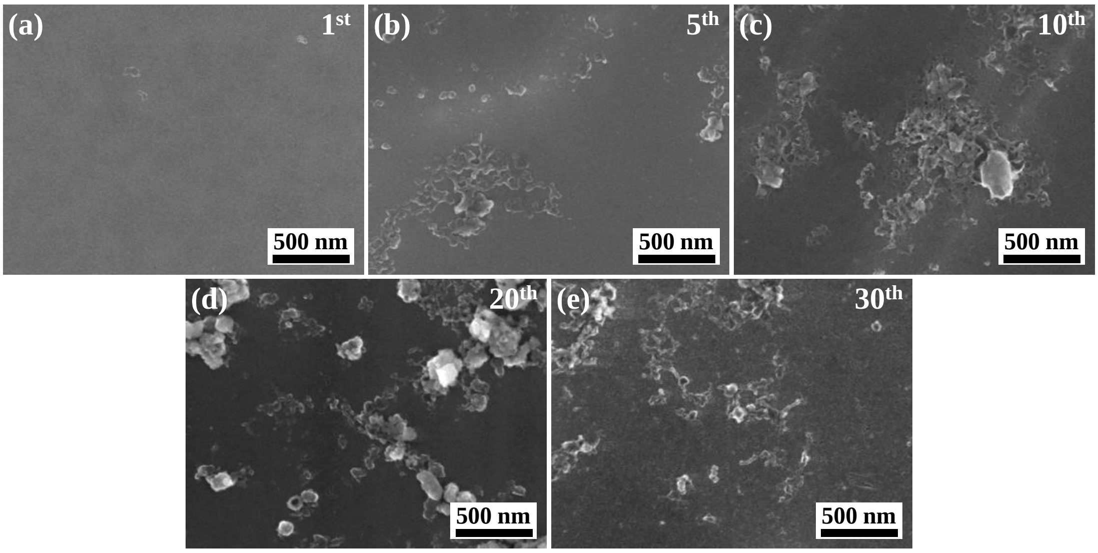

Figure S20. a-e) SEM micrographs of surface morphologies of the reused AN700 ribbons.

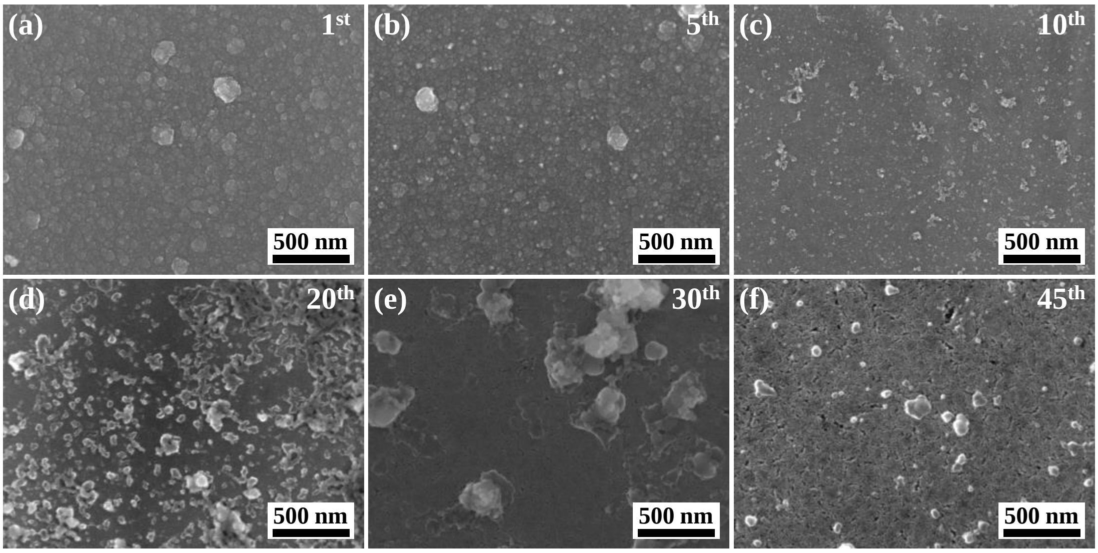

Figure S21. a-f) SEM micrographs of surface morphologies of the reused HG@AN700 ribbons.

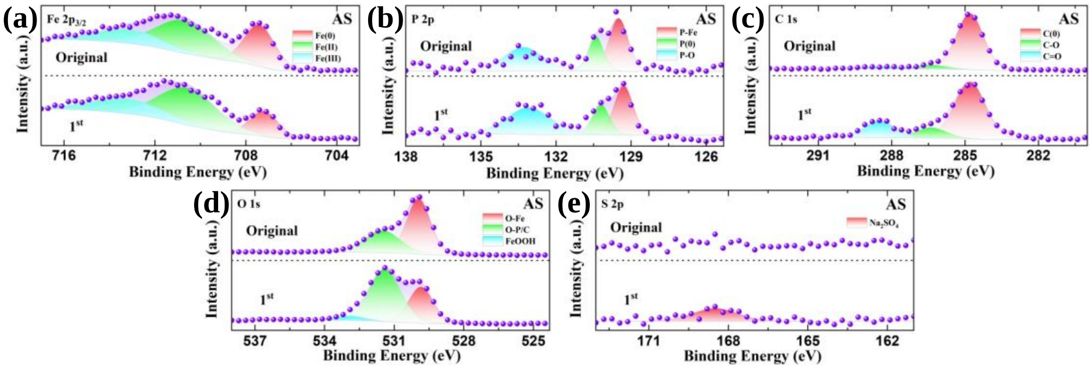

Figure S22. XPS spectra of a) Fe 2p$_{3/2}$, b) P 2p, c) C 1s, d) O 1s and e) S 2p of the original and reused AS ribbons.

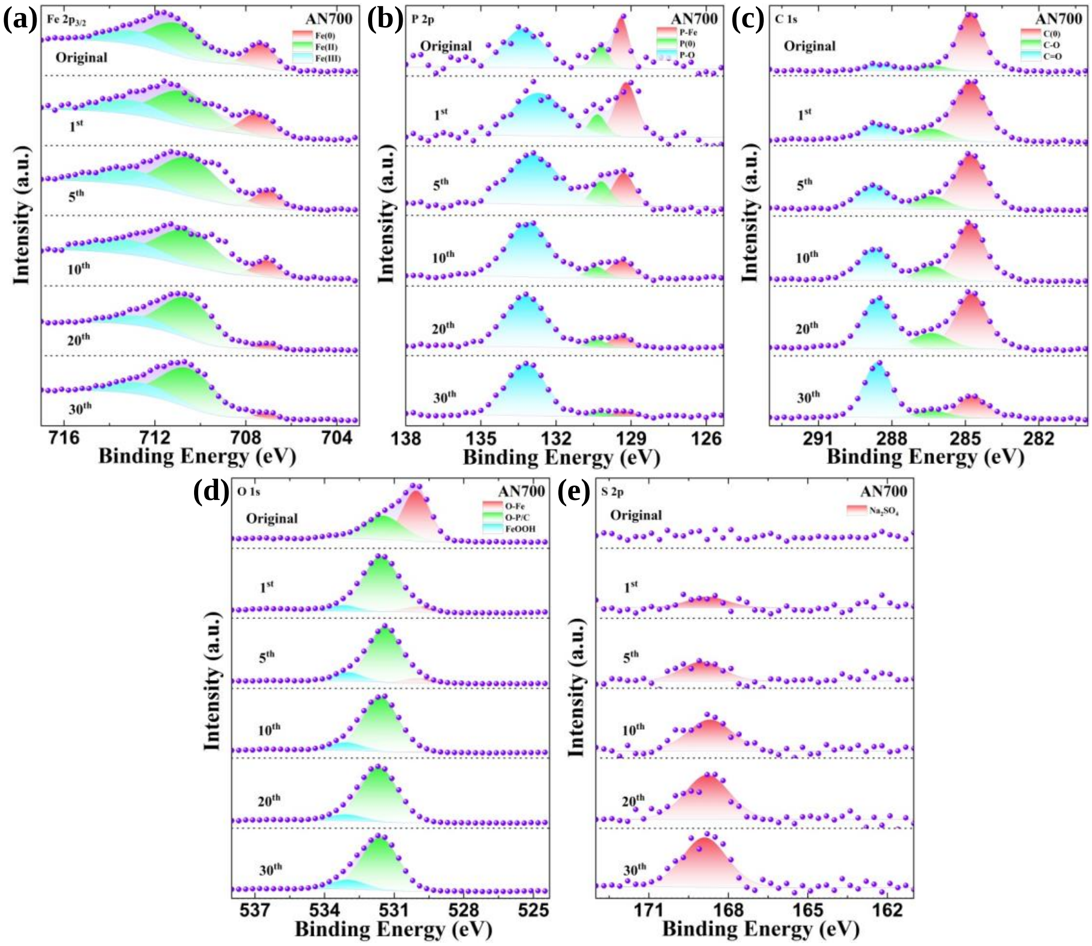

Figure S23. XPS spectra of a) Fe $2p_{3/2}$, b) P 2p, c) C 1s, d) O 1s and e) S 2p of the original and reused AN700 ribbons.

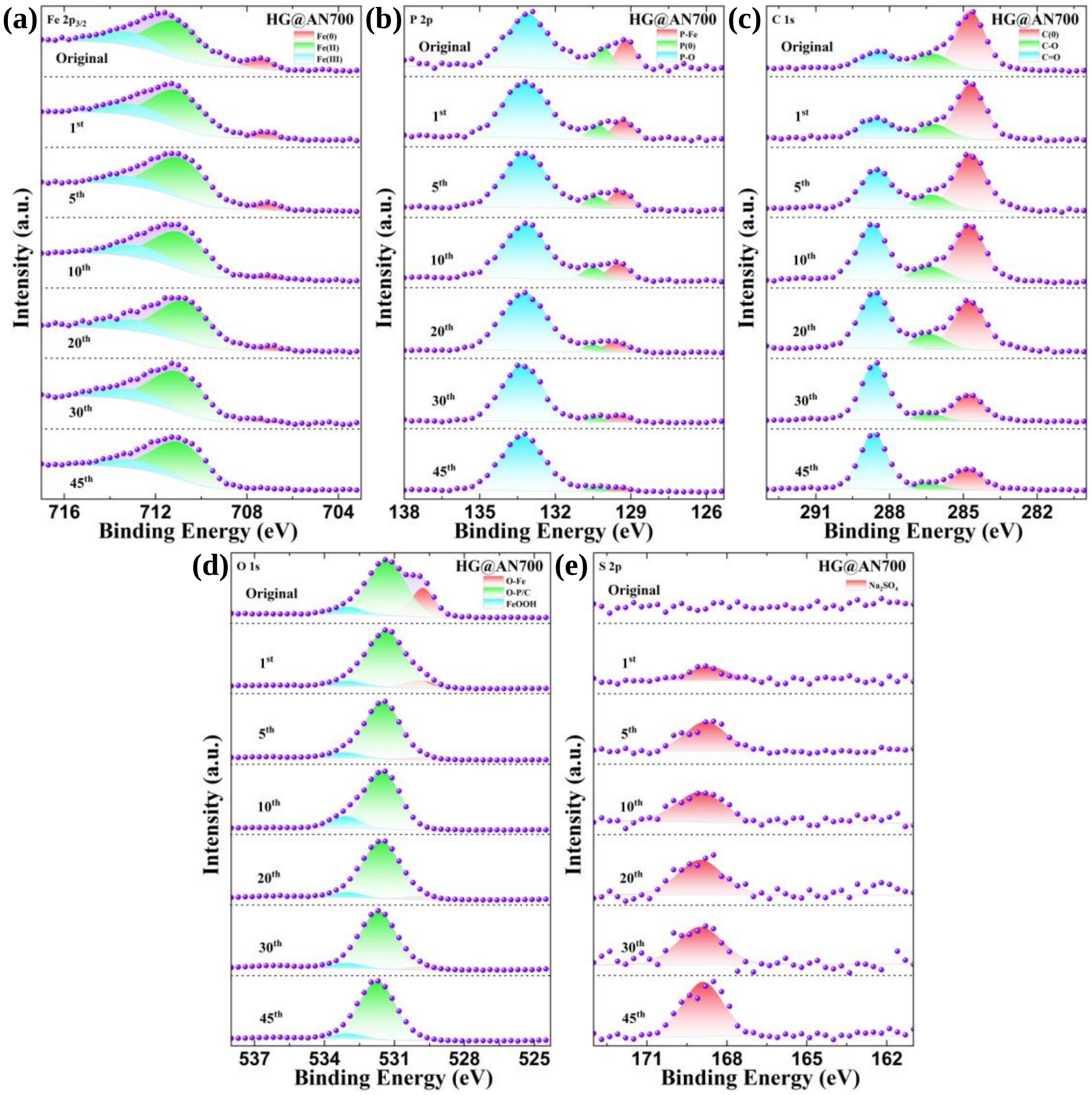

Figure S24. XPS spectra of a) Fe $2p_{3/2}$, b) P 2p, c) C 1s, d) O 1s and e) S 2p of the original and reused HG@AN700 ribbons.

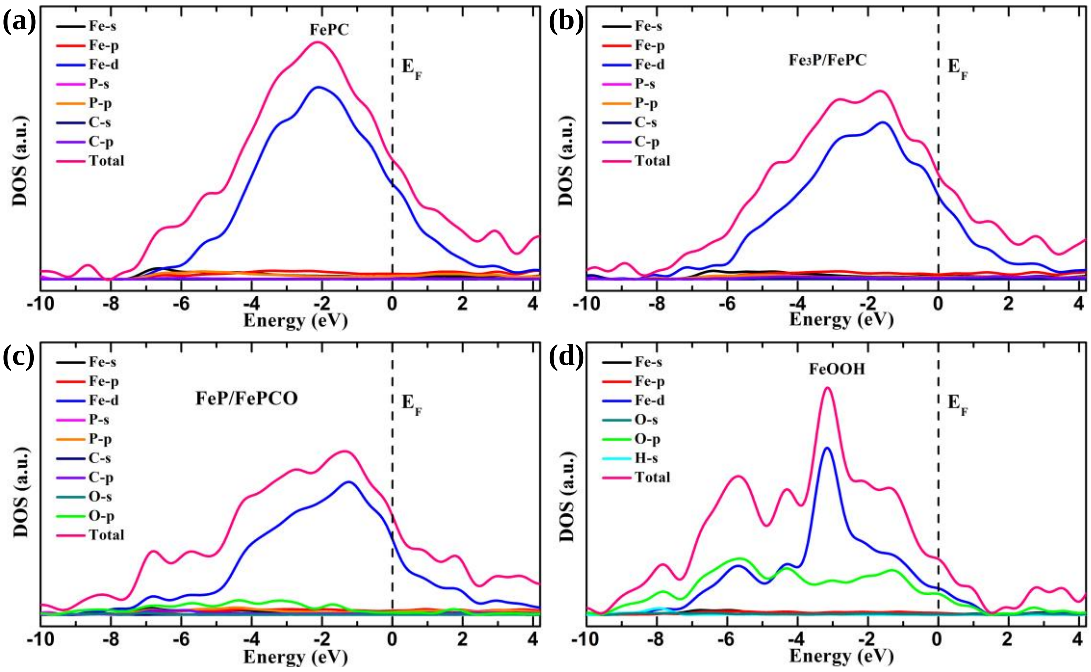

Figure S25. a-d) Total and partial DOS of the FePC, Fe_3P/FePC, FeP/FePCO and FeOOH atomic configurations, respectively, the black dashed lines at the energy of zero indicate the Fermi level.

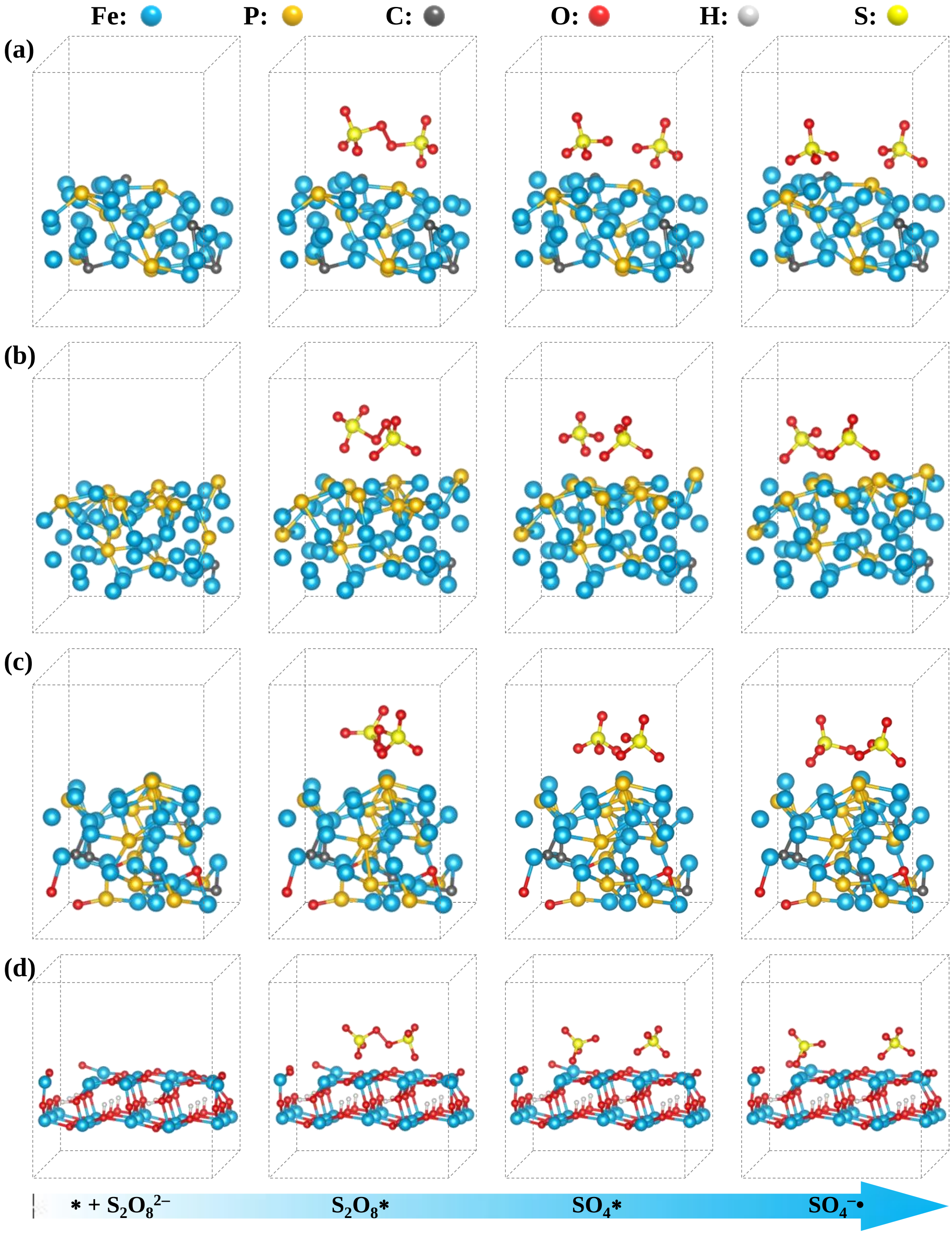

Figure S26. a-d) the FePC, Fe_3P/FePC, FeP/FePCO and FeOOH atomic configurations for $S_2O_8^{2-}$ to SO_4^{-}• conversion diagrams, respectively.

Table S1. XPS analysis of the Fe $2p_{3/2}$, P 2p, C 1s, O 1s and S 2p of the AS, AN700 and HG@AN700 ribbons in different states.

Alloy	spectrum		Original			1st		
			E (eV)	A	f_A (%)	E (eV)	A	f_A (%)
AS	Fe $2p_{3/2}$	Fe^0	707.4	11356.6	31.47	707.2	6424.3	18.57
		Fe^{2+}	710.7	16852.6	46.69	710.6	18872.9	54.57
		Fe^{3+}	713.1	7882.3	21.84	713.2	9288.8	26.86
	P 2p	P-Fe	129.5	953.9	41.50	129.3	1474.6	35.74
		P^0	130.4	486.7	21.17	130.2	803.9	19.48
		P-O	133.3	857.9	37.32	133.1	1847.5	44.78
	C 1s	C^0	284.8	26037.3	88.99	284.8	10845.1	67.88
		C-O	286.4	2115.8	7.23	286.4	1976.0	12.37
		C=O	288.4	1104.0	3.77	288.5	3156.0	19.75
	O 1s	O- Fe	530.0	46376.3	62.52	529.9	31684.2	33.57
		O-P/C	531.5	27804.2	37.48	531.4	58323.4	61.80
		FeOOH	-	-	-	532.9	4363.4	4.62
	S 2p	Na_2SO_4	-	-	-	168.5	477.3	100

Alloy	spectrum		Original			1st			5th			10th		
			E (eV)	A	f_A (%)	E (eV)	A	f_A (%)	E (eV)	A	f_A (%)	E (eV)	A	f_A (%)
AN700	Fe $2p_{3/2}$	Fe^0	707.3	6159.2	20.09	707.5	4999.4	19.95	707.0	4206.3	12.95	707.1	2769.9	12.24
		Fe^{2+}	711.0	16420.1	53.55	710.8	13579.3	54.20	710.5	19655.4	60.50	710.6	13520.5	59.75
		Fe^{3+}	712.8	8082.3	26.36	713.0	6477.1	25.85	713.1	8627.0	26.55	713.2	6337.6	28.01
	P 2p	P-Fe	129.4	528.4	28.46	129.2	466.2	30.48	129.2	1338.9	19.07	129.4	1384.9	14.21
		P^0	130.1	235.7	12.69	130.4	151.3	9.89	130.2	886.8	12.63	130.4	728.1	7.47
		P-O	133.2	1092.7	58.85	132.7	912.2	59.63	132.9	4796.1	68.30	133.1	7630.3	78.31
	C 1s	C^0	284.8	14840.3	81.32	284.8	15256.1	67.15	284.8	14545.1	58.19	284.8	13223.2	53.17
		C-O	286.4	1458.1	7.99	286.4	3014.6	13.27	286.4	3670.2	14.68	286.4	3622.4	14.57
		C=O	288.5	1951.2	10.69	288.5	4449.5	19.58	288.7	6781.1	27.13	288.7	8022.4	32.26
	O 1s	O- Fe	530.0	32906.0	59.90	530.0	6062.4	6.62	530.0	5764.6	6.51	530.0	1390.2	1.55
		O-P/C	531.5	22033.0	40.10	531.6	78397.7	85.67	531.4	72275.3	81.59	531.6	77501.0	86.56
		FeOOH	-	-	-	533.2	7054.8	7.71	533.0	10541.7	11.90	533.1	10642.4	11.89
	S 2p	Na_2SO_4	-	-	-	168.8	453.4	100	169.0	854.7	100	168.7	1456.0	100

Alloy	spectrum		20th			30th		
			E (eV)	A	f_A (%)	E (eV)	A	f_A (%)
AN700	Fe $2p_{3/2}$	Fe^0	707.1	2905.1	4.87	707.1	1848.7	4.79
		Fe^{2+}	710.5	44553.4	74.66	710.5	27293.3	70.72
		Fe^{3+}	712.6	12220.2	20.48	712.6	9450.6	24.49
	P 2p	P-Fe	129.4	1333.0	9.92	129.3	451.2	4.93
		P^0	130.4	829.4	6.17	130.3	367.5	4.01
		P-O	133.2	11271.5	83.90	133.2	8340.2	91.06
	C 1s	C^0	284.8	11842.7	44.80	284.7	6701.4	26.83

		C-O	286.4	3994.2	15.11	286.4	2634.5	10.55						
		C=O	288.6	10599.7	40.09	288.6	15645.5	62.63						
		O- Fe	-	-	-	-	-	-						
	O 1s	O-P/C	531.6	90940.8	90.17	531.6	97589.4	85.53						
		FeOOH	533.1	9917.1	9.83	533.0	16514.1	14.47						
	S 2p	Na_2SO_4	168.8	2069.0	100	168.9	2313.4	100						
Alloy	spectrum		Original			1st			5th			10th		
			E (eV)	*A*	f_A (%)	*E* (eV)	*A*	f_A (%)	*E* (eV)	*A*	f_A (%)	*E* (eV)	*A*	f_A (%)
HG@AN700	Fe $2p_{3/2}$	Fe^0	707.4	2883.2	9.06	707.2	2532.8	6.46	707.1	2766.9	6.0	707.1	1491.7	3.29
		Fe^{2+}	711.1	20520.7	64.51	711.0	26508.4	67.57	710.9	34564.9	75.01	710.9	31727.0	70.03
		Fe^{3+}	713.1	8408.3	26.43	712.9	10188.7	25.97	712.9	8751.2	18.99	712.7	12086.8	26.68
	P 2p	P-Fe	129.2	880.4	17.81	129.3	1655.5	12.84	129.4	1355.0	12.87	129.5	1818.7	11.76
		P^0	130.1	579.9	11.73	130.3	1028.8	7.98	130.4	807.6	7.67	130.5	1356.3	8.77
		P-O	133.1	3482.9	70.46	133.2	10207.0	79.18	133.2	8362.4	79.45	133.2	12296.2	79.48
	C 1s	C^0	284.7	15801.7	60.86	284.7	11672.3	58.16	284.7	10812.5	50.50	284.8	11799.5	45.08
		C-O	286.3	4680.8	18.03	286.2	3247.9	16.18	286.3	2964.7	13.85	286.4	3520.2	13.45
		C=O	288.4	5480.8	21.11	288.5	5147.9	25.65	288.5	7634.7	35.66	288.7	10853.9	41.47
	O 1s	O- Fe	529.8	21006.5	22.54	529.9	10333.7	9.48	530.0	3320.7	3.23	-	-	-
		O-P/C	531.3	65473.0	70.25	531.4	92080.2	84.44	531.5	91939.4	89.48	531.5	91169.0	85.52
		FeOOH	533.0	6724.0	7.21	533.0	6640.0	6.09	533.1	7485.2	7.29	533.1	15442.7	14.48
	S 2p	Na_2SO_4	-	-	-	168.7	802.9	100	168.8	1785.3	100	169.0	2142.0	100
Alloy	spectrum		20th			30th			45th					
			E (eV)	*A*	f_A (%)	*E* (eV)	*A*	f_A (%)	*E* (eV)	*A*	f_A (%)			
HG@AN700	Fe $2p_{3/2}$	Fe^0	707.0	1377.6	3.55	707.3	1026.4	2.67	-	-	-			
		Fe^{2+}	710.7	28663.6	73.77	711.0	28654.6	74.64	710.9	45014.5	79.37			
		Fe^{3+}	713.0	8814.6	22.69	713.0	8709.3	22.69	712.9	11703.6	20.63			
	P 2p	P-Fe	129.6	1492.0	8.16	129.5	607.8	5.79	129.4	649.8	3.75			
		P^0	130.5	885.8	4.85	130.4	375.8	3.58	130.4	629.6	3.63			
		P-O	133.2	15904.4	86.99	133.3	9512.3	90.63	133.3	16062.3	92.62			
	C 1s	C^0	284.8	10029.0	40.47	284.8	10204.0	31.29	284.8	7109.7	26.35			
		C-O	286.4	3329.0	13.43	286.4	2754.0	8.45	286.5	2389.7	8.86			
		C=O	288.6	11421.9	46.09	288.6	19649.1	60.26	288.6	17485.3	64.80			
	O 1s	O- Fe	530.0	5196.8	4.53	530.0	4521.0	4.22	530.0	1885.3	1.70			
		O-P/C	531.5	102438.9	89.32	531.6	95628.3	89.22	531.7	101485.3	91.56			
		FeOOH	533.0	7056.1	6.15	533.0	7028.3	6.56	533.0	7467.0	6.74			
	S 2p	Na_2SO_4	169.0	2371.3	100	169.0	2557.5	100	168.9	3318.2	100			

Table S2 Parameters from EIS measurements: solution resistance (R_s), resistance of passivation film (C_{f1} and R_{f1}, Q_{f2} and R_{f2}), resistance of charge transfer (C_{ct} and R_{ct}), total resistance (R_{total}) of the ribbons.

Alloy	R_s ($\Omega\cdot cm^2$)	C_{f1} ($10^{-8}\Omega^{-1}$ cm^{-2})	R_{f1} ($\Omega\cdot cm^2$)	Q_{f2}		R_{f2} ($\Omega\cdot cm^2$)	C_{ct} (Ω^{-1} cm^{-2})	R_{ct} ($\Omega\cdot cm^2$)	R_{total} ($\Omega\cdot cm^2$)	Chi-square χ^2
				Y_{f2} ($10^{-3}\Omega^{-1}s^{-n}$ cm^{-2})	N_{f2}					
AS	7.72	8.86	91.51	1.27	0.70	0.005	2.22×10^{-14}	2402.4	2501.6	0.0030
AN700	4.56	11.9	51.51	2.46	0.31	13.9	1.04×10^{-3}	1330.6	1400.6	0.0021
HG@AN700	7.18	10.2	86.51	0.95	0.80	1121.8	0.031	668.5	1884.0	0.0013

Table S3 Comparison of activation energy (ΔE) for various metallic glass, crystal and oxide environmental catalysts.

Catalysts (Metallic glass)	Organic pollutants	Activation energy (ΔE) (kJ/mol)	Ref.
$Fe_{84}B_{16}$	Direct blue 6	25.43	[7]
$Fe_{80}B_{20}$	Direct blue 15	37.4/49.1	[8]
$Fe_{80}Si_9B_{11}$	Rhodamine B	30.4	[9]
$Fe_{78}Si_{13}B_9$	Direct blue 6	17.98	[10]
$Fe_{78}Si_{13}B_9$	Rhodamine B	27.4	[11]
$Fe_{78}Si_9B_{13}$	Methylene blue	34.8	[12]
$Fe_{78}Si_9B_{13}$	Rhodamine B	27.9	[9]
$Fe_{78}Si_9B_{13}$	Methylene blue	43	[13]
$Fe_{78}(Si,B)_{22}$	Orange II	27.9	[14]
$Fe_{78}Si_8B_{14}$	Acid orange II	27.9	[15]
$Fe_{72}Si_2B_{20}Nb_6$	Direct blue 15	34.6	[16]
$Fe_{73}Si_7B_{17}Nb_3$	Direct blue 6	78	[17]
$Fe_{76}B_{12}Si_9Y_3$	Methyl orange	22.6	[18]
$Fe_{81}Si_4B_{14}Cu_1$	Orange II	20.3	[19]
$Fe_{81}Si_4B_{14}Cu_1$	Methylene blue	34.2	[19]
$Fe_{76}Si_9B_{10}P_5$	Methyl orange	24.2	[20]
$Fe_{76}Si_9B_{10}P_5$	Direct blue 6	20.4	[20]
$Fe_{68}Co_{10}Si_8B_{14}$	Acid orange II	18.4	[21]
$(Fe_{0.99}Mo_{0.01})_{78}Si_9B_{13}$	Acid orange II	28.4	[22]
$Fe_{83}Si_2B_{11}P_3C_1$	Rhodamine B	29.3	[23]
$Fe_{81}Si_2B_{10}P_6Cu_1$	Methylene blue	31	[13]
$Fe_{73.5}Si_{13.5}B_9Cu_1Nb_3$	Eosin Y	22.2	[24]
$Fe_{73.5}Si_{13.5}B_9Cu_1Nb_3$	Rhodamine B	37.49	[25]
$Fe_{80}P_{13}C_7$	Methylene blue	22.8	[12]
$Fe_{75}P_{15}C_{10}$	Reactive red 195	26.36	This work
$Fe_{75}P_{15}C_{10}$	Reactive red 195	18.37	This work
$Fe_{50}Ni_{30}P_{13}C_7$	Brilliant black BN	46.6/36.7	[26]
$Fe_{81}B_{10}P_9$	Methylene blue	36.9	[27]
$Fe_{63}Cr_5Nb_4Y_6B_{22}$	Methylene blue	21.8	[28]
$Fe_{36}Co_{36}Si_{4.8}B_{19.2}Nb_4$	Rhodamine B	33.35	[25]
$Fe_{70}Cr_5Ni_3Mo_3W_9Si_5B_5$	Blliant red 3B-A	46.5	[29]
$Co_{78}Si_8B_{14}$	Acid orange II	33	[30]
$Co_{65}Mo_{15}B_{20}$	Direct blue 6	20.5	[31]
$Cu_{46}Zr_{44.5}Al_{7.5}Gd_2$	Acid orange II	17	[32]
$Al_{85}Ni_9Y_6$	Direct blue 2B	29/40/46	[33]
$Al_{85}Fe_{10}Y_5$	Methyl orange	23.8	[34]
$Al_{85}Co_{10}Y_5$	Methyl orange	21.3	[34]
$Mg_{73}Zn_{21.5}Ca_{5.5}$	Direct blue 6	51	[35]

Catalysts (Metallic crystal)	Organic pollutants	Activation energy (Δ*E*) (kJ/mol)	Ref.
NZVI particles	Methyl orange	35.9	[36]
Fe powder	Direct blue 6	31.98	[7]
Fe powder	Methyl orange	48.3	[20]
Fe powder	Direct blue 6	34.8	[20]
Fe plate	Orange II	30.79/35.90/40.0	[37]
$(Fe_{0.99}Mo_{0.01})_{78}Si_9B_{13}$	Acid orange II	28.2	[22]
$Fe_{76}Si_9B_{10}P_5$	Direct blue	19.5	[38]
$Fe_{76}Si_9B_{10}P_5$	Methyl orange	26.8	[38]
$Fe_{81}Si_2B_{10}P_6Cu_1$	Methylene blue	27/43	[13]
$Zr_{55}Cu_{30}Ni_5Al_{10}$	Methyl orange	30.6	[39]
AlCoCrTiZn	Direct blue 6	30	[40]
H-Fe-P-B	Acid light yellow G	31.0	[41]
Fe-Carbon aerogel	Orange II	56.1	[42]
**Catalysts (Oxide)**	**Organic pollutants**	**Activation energy (Δ*E*) (kJ/mol)**	**Ref.**
MnO_2	Phenol	21.9	[43]
Mn_2O_3	Phenol	61.2	[44]
Mn_3O_4	Phenol	38.5	[45]
Co_3O_4	Phenol	66.2	[45]
Co_3O_4/RM	Phenol	46.2	[46]
Co_3O_4/SBA-15	Phenol	81.4/67.4	[47]
Co_3O_4/SiO_2–N	Phenol	67.5/75.1/61.7	[48]
Co_3O_4-ZSM-5	Phenol	69.7	[49]
Co_2O_3/AC	Phenol	59.7	[50]
Co_3O_4/FA	Phenol	47.0/56.5/56.0	[51]
Co_3O_4/CA	Phenol	62.9	[52]
Co_3O_4–rGO	Phenol	26.5	[53]
Mn_3O_4–rGO	Orange II	49.5	[54]
Co_3O_4/CX	Phenol	62.9/48.3	[55]
Fe_2O_3-ZSM-5	M-cresol	81.3	[56]
Ordinary thermal reactions	-	60-250	[41]

Table S4 Comparison of total organic carbon (TOC) removal for various metallic glass, crystal and oxide environmental catalysts.

Catalysts (Metallic glass)	Organic pollutants	Initial concentration (mg L^{-1})	Reaction time (min)	TOC removal (%)	Ref.
$Fe_{78}Si_9B_{13}$	Rhodamine B	20	30	55	[9]
$Fe_{80}Si_9B_{11}$	Rhodamine B	20	30	45	[9]
$Fe_{78}Si_9B_{13}$	Methyl blue	20	20	52	[57]
$Fe_{78}Si_9B_{13}$	Methyl orange	20	20	61	[57]
$Fe_{78}Si_9B_{13}$	Brilliant red 3B-A	20	60	83.6	[58]
$Fe_{78}Si_9B_{13}$	Methylene blue	20	10	54.4	[59]
$Fe_{81}Si_4B_{14}Cu_1$	Orange II	100	50	16/23/15/17/31	[60]
$Fe_{81}Si_2B_{10}P_6Cu_1$	Methylene blue	100	15	32	[13]
$Fe_{83}Si_2B_{11}P_3C_1$	Rhodamine B	20	30	52	[23]
$Fe_{83}Si_2B_{11}P_3C_1$	Methylene blue	20	30	60	[23]
$Fe_{83}Si_2B_{11}P_3C_1$	Methyl orange	20	30	62	[23]
$Fe_{83}Si_2B_{11}P_3C_1$	Mixed dyes	20	30	55	[23]
$Fe_{73.5}Si_{13.5}B_9Cu_1Nb_3$	Methyl blue	20	20	45	[57]
$Fe_{73.5}Si_{13.5}B_9Cu_1Nb_3$	Methyl orange	20	20	51	[57]
$Fe_{73.5}Si_{13.5}B_9Cu_1Nb_3$	Methylene blue	20	30	38/50	[61]
$Fe_{73.5}Si_{13.5}B_9Cu_1Nb_3$	Methylene blue	20	45	44.6	[59]
$Fe_{80}B_{13}C_7$	Acid orange 7	20	120	55	[62]
$Fe_{75}P_{15}C_{10}$	Reactive red 195	100	30	79.5	This work
$Fe_{75}P_{15}C_{10}$	Reactive black 5	100	30	82.6	This work
$Fe_{75}P_{15}C_{10}$	Rhodamine B	100	30	84.3	This work
$Fe_{75}P_{15}C_{10}$	Mixed dyes	100	30	80.7	This work
$Fe_{50}Ni_{30}P_{13}C_7$	Brilliant black BN	20	20	23.4/45.1	[26]
$Cu_{46}Zr_{44.5}Al_{7.5}Gd_2$	Acid orange II	100	30	74	[32]
$Co_{78}Si_8B_{14}$	Acid orange II	100	15	80.2/74.6	[63]
$Co_{78}Si_8B_{14}$	Rhodamine B	100	40	52/48	[63]
$Al_{85}Fe_{10}Y_5$	Methyl orange	10	45	75.1	[34]
$Al_{85}Co_{10}Y_5$	Methyl orange	10	45	82.4	[34]
**Catalysts (Metallic crystal)**	**Organic pollutants**	**Initial concentration (mg L^{-1})**	**Reaction time (min)**	**TOC removal (%)**	**Ref.**
NZVI	Acid black 24	100	10	53.8	[64]
$Fe_{78}Si_9B_{13}$	Methylene blue	20	20	46.1	[59]
$Fe_{73.5}Si_{13.5}B_9Cu_1Nb_3$	Methylene blue	20	45	35.4	[59]
FeCuC-aerogel	Methylene blue	50	60	82	[65]
Fe-AC	M-cresol	100	120	83	[66]
H-Fe-P-B	Acid light yellow G	50	120	65	[41]
H-Fe-S	Rhodamine B	95.8	100	38	[67]
H-Fe-S	4-Nitrophenol	50	150	80	[67]
**Catalysts (Oxide)**	**Organic pollutants**	**Initial concentration (mg L^{-1})**	**Reaction time (min)**	**TOC removal (%)**	**Ref.**
Mn_2O_3	Phenol	25	60	90.5	[44]
Mn_2O_3	Phenol	25	120	86.39	[68]
Mn_3O_4	Phenol	25	60	50	[45]
Co_3O_4	Phenol	5	60	68	[45]
Co_3O_4/RM-T	Phenol	25	60	57/64	[46]
Co_2O_3/AC	Phenol	25	60	80	[50]
Fe_3O_4@OMC/CA	Dimethyl phthalate	50	120	65	[69]
FeOOH/γ-Al_2O_3	Rhodamine B	10	150	78.82/69.64	[70]

$CuBi_2O_4/Bi_3ClO_4$	Acid brown 14	10	120	75	[71]
Fe–Co/SBA	Orange II	105	120	33.8	[72]
CoFe–LDH/CF	Acid orange II	40	120	87	[73]

Table S5 Comparison of catalytic efficiency and reusability for various metallic glass, crystal and oxide environmental catalysts.

Catalysts (Metallic glass)	Organic pollutants	Initial concentration (mg L^{-1})	Area dosage (m^2 L^{-1})	k (min^{-1})	k_{SA} (L m^{-2} min^{-1})	$k_{SA} \cdot C_0$ (mg m^{-2} min^{-1})	Reusability (times)	Ref.
$Fe_{84}B_{16}$	Direct blue 6	200	0.112	0.110	0.982	196.4	-	[7]
$Fe_{82}B_{18}$	Direct blue 6	200	0.125	0.113	0.904	180.8	-	[7]
$Fe_{80}B_{20}$	Direct blue 6	200	0.160	0.156	0.975	195	-	[7]
$Fe_{80}B_{20}$	Direct blue 15	200	0.155	0.228	1.47	294	5	[8]
$Fe_{78}Si_9B_{13}$	Methyl orange	20	0.06	0.386	6.43	128.6	4	[57]
$Fe_{78}Si_9B_{13}$	Methyl blue	20	0.06	0.381	6.35	127	4	[57]
$Fe_{78}Si_9B_{13}$	Blliant red 3B-A	50	0.26	0.654	2.52	126	4	[58]
$Fe_{78}Si_9B_{13}$	Methyl blue	20	0.06	0.356	5.93	118.6	-	[74]
$Fe_{78}Si_9B_{13}$	Brilliant Yellow 3G-P	20	0.06	0.466	7.76	155.2	-	[74]
$Fe_{78}Si_9B_{13}$	Brilliant red 3B-A	20	0.06	0.372	6.2	124	-	[74]
$Fe_{78}Si_9B_{13}$	Malachite green	20	0.06	0.519	8.65	173	-	[74]
$Fe_{78}Si_9B_{13}$	Malachite green	20	0.06	0.057	0.95	19	-	[74]
$Fe_{78}Si_9B_{13}$	Acid orange II	300	-	0.15	-	-	10	[75]
$Fe_{78}Si_9B_{13}$	Methylene blue	20	0.06	0.64	10.67	213.4	20	[76]
$Fe_{78}Si_9B_{13}$	Phenol	1000	-	-	-	-	8	[77]
$Fe_{78}Si_9B_{13}$	Acid orange II	200	-	0.32	-	-	7	[78]
$Fe_{78}Si_9B_{13}$	Methylene blue	100	-	0.37	-	-	12	[12]
$Fe_{78}Si_{13}B_9$	Direct blue 6	200	0.1729	0.115	0.66	132	-	[10]
$Fe_{78}Si_{13}B_9$	Orange II	100	0.52	0.238	0.46	46	-	[10]
$Fe_{78}Si_{13}B_9$	Methyl orange	25	0.13	0.103	0.79	19.75	-	[10]
$Fe_{78}Si_9B_{13}$	Rhodamine B	20	-	0.725	-	-	4	[11]
$Fe_{78}Si_9B_{13}$	Methylene blue	20	0.06	0.302	5.03	100.6	5	[59]
$Fe_{78}(Si,B)_{22}$	Orange II	100	0.062	0.125	2.0	200	-	[14]
$Fe_{78}Si_8B_{14}$	Acid orange II	200	0.083	0.174	2.09	418	4	[15]
$Fe_{79}B_{16}Si_5$	Orange G	100	0.005	0.004	0.8	80	8	[79]
$Fe_{66.3}B_{16.6}Y_{17.1}$	Orange G	100	0.005	0.047	9.4	940	11	[79]
$Fe_{76}B_{12}Si_9Y_3$	Methyl orange	20	-	-	-	-	13	[18]
$Fe_{78}Si_{11}B_9P_2$	Orange II	20	-	0.661	-	-	17	[80]
$Fe_{81}Si_4B_{14}Cu_1$	Orange II	100	-	0.09	-	-	45	[19]
$(Fe_{0.99}Mo_{0.01})_{78}Si_9B_{13}$	Acid orange II	100	0.083	0.168	2.016	201.6	-	[22]
$(Fe_{0.99}Mo_{0.01})_{78}Si_9B_{13}$	Direct blue 2B	200	-	0.136	-	-	4	[81]
$Fe_{73.5}Si_{13.5}B_9Cu_1Nb_3$	Methyl orange	20	0.06	0.152	2.53	50.6	4	[57]
$Fe_{73.5}Si_{13.5}B_9Cu_1Nb_3$	Methyl blue	20	0.06	0.201	3.35	67	4	[57]
$Fe_{73.5}Si_{13.5}B_9Cu_1Nb_3$	Methylene blue	20	0.06	0.119	1.98	39.6	5	[59]
$Fe_{73.5}Si_{13.5}B_9Cu_1Nb_3$	Methylene blue	20	-	0.064	-	-	30	[61]
$Fe_{83}Si_2B_{11}P_3C_1$	Rhodamine B	20	0.002	0.09	45	900	35	[23]
$Fe_{83}Si_2B_{11}P_3C_1$	Rhodamine B	20	0.002	0.36	171.43	3428.6	-	[23]
$Fe_{83}Si_2B_{11}P_3C_1$	Rhodamine B	20	0.002	0.165	78.57	1571.4	-	[23]
$Fe_{81}Si_2B_{10}P_6Cu_1$	Methylene blue	100	-	0.58	-	-	20	[13]
$Fe_{80}B_{13}C_7$	Acid orange 7	20	-	0.08	-	-	22	[62]
$Fe_{80}P_{13}C_7$	Acid orange 7	20	-	0.04	-	-	5	[62]
$Fe_{81}B_{10}C_9$	Methylene blue	20	-	0.18	-	-	15	[27]
$Fe_{81}B_{10}P_9$	Methylene blue	20	-	0.13	-	-	10	[27]
$Fe_{80}P_{13}C_7$	Methylene blue	100	-	0.56	-	-	23	[12]

$Fe_{75}P_{15}C_{10}$	Reactive red 195	100	0.0097	0.111	11.44	1144	-	This work
$Fe_{75}P_{15}C_{10}$	Reactive red 195	100	0.0097	0.295	30.41	3041	24	This work
$Fe_{75}P_{15}C_{10}$	Reactive red 195	100	0.0099	0.307	31.01	3101	39	This work
$Fe_{63}Cr_5Nb_4Y_6B_{22}$	Methylene blue	100	-	0.182	-	-	3	[28]
$Fe_{79.2}Co_4P_{10}C_6Cu_{0.8}$	Methylene blue	100		0.470			13	[82]
$Fe_{36}Co_{36}Si_{4.8}B_{19.2}Nb_4$	Rhodamine B	20	-	0.06	-	-	4	[25]
$Fe_{70}Cr_5Ni_3Mo_3W_9Si_5B_5$	Blliant red 3B-A	20		0.586			45	[29]
CS1-CS3	Orange II	25		0.094-0.351			100	[83]
$Cu_{47.5}Zr_{46}Al_{6.5}$	Acid orange II	100	1.0	0.165	0.165	16.5	10	[84]
$Cu_{46}Zr_{44.5}Al_{7.5}Gd_2$	Acid orange II	100	-	0.26	-	-	80	[32]
$Co_{78}Si_8B_{14}$	Acid orange II	100	-	0.089	-	-	25	[63]
$Co_{78}Si_8B_{14}$	Acid orange II	100	-	0.176	-	-	60	[63]
$Co_{78}Si_8B_{14}$	Acid orange II	200	1.44	2.687	1.866	373.2	8	[30]
$Co_{65}Mo_{15}B_{20}$	Direct blue 6	20	-	2.31	-	-	20	[31]
$Mg_{65}Cu_{25}Y_{10}$	Direct blue 6	20	37.61	0.585	0.016	0.32	-	[85]
$Mg_{65}Cu_{25}Y_{10}$	Direct blue 6	20	19.94	0.222	0.011	0.22	-	[85]
**Catalysts (Metallic crystal)**	**Organic pollutants**	**Initial concentration (mg L^{-1})**	**Area dosage (m^2 L^{-1})**	**k (min^{-1})**	**k_{SA} (L m^{-2} min^{-1})**	**k_{SA} C_0 (mg m^{-2} min^{-1})**	**Reusability (times)**	**Ref.**
NZVI	Methyl orange	50	23.2	0.559	0.024	1.2	-	[36]
NZVI	Acid black 24	100	23.2	0.199	0.0086	0.86	-	[64]
Micro-ZVI	Orange II	105	1.42	0.38	0.268	28.14	-	[86]
Micro-ZVI	Orange II	150.5	-	0.022	-	-	-	[87]
Micro-ZVI	Orange II	49	-	0.017	-	-	-	[87]
Fe powder	Direct blue 6	200	4.573	0.049	0.011	2.2	-	[7]
Fe plate	Orange II	35	-	0.00742	0.18	6.3	3	[37]
$Fe_{80}B_{20}$	Direct blue 6	200	0.112	0.062	0.554	110.8	-	[7]
$Fe_{78}Si_9B_{13}$	Methylene blue	20	0.06	0.258	4.30	86	5	[59]
$Fe_{73.5}Si_{13.5}B_9Cu_1Nb_3$	Methylene blue	20	0.06	0.111	1.85	37	5	[59]
$(Fe_{73.5}Si_{13.5}B_9Nb_3Cu_1)_{91.5}Ni_{8.5}$	Orange II	25	0.139	0.160	1.15	28.75	9	[88]
$(Fe_{0.99}Mo_{0.01})_{78}Si_9B_{13}$	Acid orange II	100	0.083	0.069	0.828	82.8	-	[22]
Fe-ABs	Indole	20	1.864	0.0678	0.036	0.72	4	[89]
$Zr_{55}Cu_{30}Ni_5Al_{10}$	Methyl orange	20	163.2	0.147	9.0×10^{-4}	0.018	5	[39]
$Mn_{70}Al_{30}$	Orange II	40	0.565	0.091	0.161	6.44	4	[90]
**Catalysts (Oxide)**	**Organic pollutants**	**Initial concentration (mg L^{-1})**	**Area dosage (m^2 L^{-1})**	**k (min^{-1})**	**k_{SA} (L m^{-2} min^{-1})**	**k_{SA} C_0 (mg m^{-2} min^{-1})**	**Reusability (times)**	**Ref.**
Fe_3O_4	Rhodamine B	10	0.003	0.019	6.33	63.3	6	[91]
FeOOH/γ-Al_2O_3	Rhodamine B	10	6670	0.1055	1.58×10^{-5}	1.58×10^{-4}	5	[70]
Fe_3O_4/rGO	Reactive red 195	168.43	-	0.044	-	-	6	[92]
Fe_2O_3-ZSM-5	M-cresol	1000	629.25	0.0125	1.99×10^{-5}	0.019	-	[56]
Fe–Co/SBA	Orange II	105	213.438	0.0246	1.15×10^{-4}	0.012	4	[72]
CoFe–LDH/CF	Acid orang II	40	-	-	-	-	7	[73]
MNPs@C	Industrial wastewater	800	671.2	0.0513	0.000076	0.0608	5	[93]

1. Area dosage (ρ) is calculated from $\rho = S \cdot V^{-1}$, S and V demonstrate the specific surface area of the catalysts and the volume of the dye solution.

2. Essential dye degradation surface area normalized rate constant (k_{SA}) is calculated from the kinetic rate (k) by

dividing the area dosage (ρ), whereas the k is based on first kinetic model: $\ln(C_0/C_t) = k\,t$, C_0 is the initial concentration of dye at t = 0; C_t is the dye concentration at time t.

3. The Supplementary References

[1] G. Kresse, J. Furthmüller, Comput. Mater. Sci. 6 (1996) 15-50.

[2] J.P. Perdew, K. Burke, M. Ernzerhof, Phys. Rev. Lett. 77 (1996) 3865.

[3] G. Kresse, J. Furthmüller, Phys. Rev. B 54 (1996) 11169-11186.

[4] H.J. Monkhorst, J.D. Pack, Phys. Rev. B 13 (1976) 5188-5192.

[5] S. Grimme, J. Antony, S. Ehrlich, H. Krieg, J. Chem. Phys. 132 (2010) 154104.

[6] G. Henkelman, B.P. Uberuaga, H. Jónsson, J. Chem. Phys. 113 (2000) 9901-9904.

[7] Y. Tang, Y. Shao, N. Chen, K.F. Yao, RSC Adv. 5 (2015) 6215-6221.

[8] R. Li, X.J. Liu, H. Wang, Y. Wu, K.C. Chan, Z.P. Lu, Mater. Des. 155 (2018) 346-351.

[9] Z. Jia, J.L. Jiang, L. Sun, L.C. Zhang, Q. Wang, S.X. Liang, P. Qin, D.F. Li, J. Lu, J.J. Kruzic, ACS Appl. Mater. Interfaces 12 (2020) 44789-44797.

[10] Y. Tang, Y. Shao, N. Chen, X. Liu, S.Q. Chen, K.F. Yao, RSC Adv. 5 (2015) 34032-34039.

[11] X. Wang, Y. Pan, Z. Zhu, J. Wu, Chemosphere 117 (2014) 638-643.

[12] Q. Wang, M. Chen, P. Lin, Z. Cui, C. Chu, B. Shen, J. Mater. Chem. A 6 (2018) 10686-10699.

[13] Q. Wang, L. Yun, M. Chen, D. Xu, Z. Cui, Q. Zeng, P. Lin, C. Chu, B. Shen, ACS Appl. Nano Mater. 2 (2018) 214-227.

[14] C. Zhang, Z. Zhu, H. Zhang, Z. Hu, Chin. Sci. Bull. 56 (2011) 3988-3992.

[15] C. Zhang, Z. Zhu, H. Zhang, Z. Hu, J. Environ. Sci. 24 (2012) 1021-1026.

[16] Z. Deng, X.H. Zhang, K.C. Chan, L. Liu, T. Li, Chemosphere 174 (2017) 76-81.

[17] J.Q. Wang, Y.H. Liu, M.W. Chen, G.Q. Xie, D.V. Louzguine Luzgin, A. Inoue, J.H. Perepezko, Adv. Funct. Mater. 22 (2012) 2567-2570.

[18] S. Xie, P. Huang, J.J. Kruzic, X. Zeng, H. Qian, Sci. Rep. 6 (2016) 21947.

[19] S.Q. Chen, K.Z. Hui, L.Z. Dong, Z. Li, Q.H. Zhang, L. Gu, W. Zhao, S. Lan, Y. Ke, Y. Shao, H. Hahn, K.F. Yao, Sci. China Mater. 63 (2019) 453-466.

[20] F. Wang, H. Wang, H. Zhang, Z. Dan, N. Weng, W. Tang, F. Qin, J. Non-Cryst. Solids 491 (2018) 34-42.

[21] C. Zhang, Z. Zhu, H. Zhang, J. Phys. Chem. Solids 110 (2017) 152-160.

[22] C. Zhang, Z. Zhu, H. Zhang, Z. Hu, J. Non-Cryst. Solids 358 (2012) 61-64.

[23] Z. Jia, Q. Wang, L. Sun, Q. Wang, L.C. Zhang, G. Wu, J.H. Luan, Z.B. Jiao, A. Wang, S.X. Liang, M. Gu, J. Lu, Adv. Funct. Mater. 29 (2019) 1807857.

[24] J.C. Wang, Z. Jia, S.X. Liang, P. Qin, W.C. Zhang, W.M. Wang, T.B. Sercombe, L.C. Zhang, Mater. Des. 140 (2018) 73-84.

[25] J.L. Jiang, Z. Jia, Q. He, Q. Wang, F. Lyu, L.C. Zhang, S.X. Liang, J.J. Kruzic, J. Lu, J. Alloys Compd. 822 (2020) 153574.

[26] S.X. Liang, W. Zhang, W. Wang, G. Jia, W. Yang, L.C. Zhang, J. Phys. Chem. Solids 132 (2019) 89-98.

[27] B. Wei, X. Li, H. Sun, K. Song, L. Wang, J. Non-Cryst. Solids 575 (2022) 121212.

[28] W. Yang, Q. Wang, W. Li, L. Xue, H. Liu, J. Zhou, J. Mo, B. Shen, Mater. Des. 161 (2019) 136-146.

[29] S.X. Liang, X. Wang, W. Zhang, Y.J. Liu, W. Wang, L.C. Zhang, Appl. Mater. Today 19 (2020) 100543.

[30] X.D. Qin, Z.W. Zhu, G. Liu, H.M. Fu, H.W. Zhang, A.M. Wang, H. Li, H.F. Zhang, Sci. Rep. 5 (2015) 18226.

[31] M. Tang, L. Lai, D. Ding, T. Liu, W. Kang, N. Guo, B. Song, S. Guo, J. Non-Cryst. Solids 576 (2022) 121282.

[32] Z. Li, X. Qin, Z. Zhu, S. Zheng, H. Li, H. Fu, H. Zhang, J. Mater. Chem. A 8 (2020) 10855-10864.

[33] P. Wang, J.Q. Wang, H. Li, H. Yang, J. Huo, J. Wang, C. Chang, X. Wang, R.-W. Li, G. Wang, J. Alloys Compd. 701 (2017) 759-767.

[34] Q. Chen, Z. Yan, L. Guo, H. Zhang, L.C. Zhang, W. Wang, J. Mol. Liq. 318 (2020) 114318.

[35] J.Q. Wang, Y.H. Liu, M.W. Chen, D.V. Louzguine-Luzgin, A. Inoue, J.H. Perepezko, Sci. Rep. 2 (2012) 418.

[36] J. Fan, Y. Guo, J. Wang, M. Fan, J. Hazard. Mater. 166 (2009) 904-910.

[37] J.A. Mielczarski, G.M. Atenas, E. Mielczarski, Appl. Catal., B 56 (2005) 289-303.

[38] N. Weng, F. Wang, F. Qin, W. Tang, Z. Dan, Materials 10 (2017).

[39] C. Yang, C. Zhang, L. Liu, J. Mater. Chem. A 6 (2018) 20992-21002.

[40] Z.Y. Lv, X.J. Liu, B. Jia, H. Wang, Y. Wu, Z.P. Lu, Sci. Rep. 6 (2016) 34213.

[41] J. Chen, L. Zhu, Catal. Today 126 (2007) 463-470.

[42] J.H. Ramirez, F.J. Maldonado-Hódar, A.F. Pérez-Cadenas, C. Moreno-Castilla, C.A. Costa, L.M. Madeira, Appl. Catal., B 75 (2007) 312-323.

[43] E. Saputra, S. Muhammad, H. Sun, H.M. Ang, M.O. Tade, S. Wang, Environ. Sci. Technol. 47 (2013) 5882-5887.

[44] E. Saputra, S. Muhammad, H. Sun, H.M. Ang, M.O. Tadé, S. Wang, Appl. Catal., B 154-155 (2014) 246-251.

[45] E. Saputra, S. Muhammad, H. Sun, H.M. Ang, M.O. Tade, S. Wang, J. Colloid. Interface Sci. 407 (2013) 467-473.

[46] S. Muhammad, E. Saputra, H. Sun, H.M. Ang, M.O. Tadé, S. Wang, Ind. Eng. Chem. Res. 51 (2012) 15351-15359.

[47] P.R. Shukla, H. Sun, S. Wang, H.M. Ang, M.O. Tadé, Catal. Today 175 (2011) 380-385.

[48] P.R. Shukla, H. Sun, S. Wang, H.M. Ang, M.O. Tadé, Sep. Purif. Technol. 77 (2011) 230-236.

[49] P.R. Shukla, S. Wang, K. Singh, H.M. Ang, M.O. Tadé, Appl. Catal., B 99 (2010) 163-169.

[50] P.R. Shukla, S. Wang, H. Sun, H.M. Ang, M. Tadé, Appl. Catal., B 100 (2010) 529-534.

[51] S. Muhammad, E. Saputra, H. Sun, J.d.C. Izidoro, D.A. Fungaro, H.M. Ang, M.O. Tadé, S. Wang, RSC Adv. 2 (2012) 5645.

[52] Y. Hardjono, H. Sun, H. Tian, C.E. Buckley, S. Wang, Chem. Eng. J. 174 (2011) 376-382.

[53] Y. Yao, Z. Yang, H. Sun, S. Wang, Ind. Eng. Chem. Res. 51 (2012) 14958-14965.

[54] Y. Yao, C. Xu, S. Yu, D. Zhang, S. Wang, Ind. Eng. Chem. Res. 52 (2013) 3637-3645.

[55] H. Sun, H. Tian, Y. Hardjono, C.E. Buckley, S. Wang, Catal. Today 186 (2012) 63-68.

[56] Y. Yang, H. Zhang, Y. Yan, R. Soc. Open Sci. 5 (2018) 171731.

[57] Z. Jia, J. Kang, W.C. Zhang, W.M. Wang, C. Yang, H. Sun, D. Habibi, L.C. Zhang, Appl. Catal., B 204 (2017) 537-547.

[58] Z. Jia, W.C. Zhang, W.M. Wang, D. Habibi, L.C. Zhang, Appl. Catal., B 192 (2016) 46-56.

[59] S.X. Liang, Z. Jia, Y.J. Liu, W.C. Zhang, W.M. Wang, J. Lu, L.C. Zhang, Adv. Mater. 30 (2018) 1802764.

[60] S.Q. Chen, M. Li, X.Y. Ma, M.J. Zhou, D. Wang, M.Y. Yan, Z. Li, K.F. Yao,

Chemosphere 264 (2021) 128392.

[61] J.C. Wang, S.X. Liang, Z. Jia, W.C. Zhang, W.M. Wang, Y.J. Liu, J. Lu, L.C. Zhang, J. Alloys Compd. 785 (2019) 642-650.

[62] F. Miao, Q. Wang, Q. Zeng, L. Hou, T. Liang, Z. Cui, B. Shen, J. Mater. Sci. Technol. 38 (2019) 107-118.

[63] X. Qin, J. Xu, Z. Zhu, Z. Li, D. Fang, H. Fu, S. Zhang, H. Zhang, J. Mater. Sci. Technol. 113 (2022) 105-116.

[64]H.Y. Shu, M.C. Chang, H.H. Yu, W.H. Chen, J. Colloid. Interface Sci. 314 (2007) 89-97.

[65] H. Zhao, L. Qian, X. Guan, D. Wu, G. Zhao, Environ. Sci. Technol. 50 (2016) 5225-5233.

[66] L. Bounab, O. Iglesias, E. González-Romero, M. Pazos, M. Ángeles Sanromán, RSC Adv. 5 (2015) 31049-31056.

[67] Y. Gao, H. Gan, G. Zhang, Y. Guo, Chem. Eng. J. 217 (2013) 221-230.

[68] E. Saputra, S. Muhammad, H. Sun, H.-M. Ang, M.O. Tadé, S. Wang, Appl. Catal., B 142-143 (2013) 729-735.

[69] Y. Wang, H. Zhao, G. Zhao, Electroanalysis 28 (2016) 169-176.

[70] G. Zhang, Y. Zhou, F. Yang, J. Electrochem. Soc. 162 (2015) H357-H365.

[71] H. Najafian, F. Manteghi, F. Beshkar, M. Salavati-Niasari, J. Hazard. Mater. 361 (2019) 210-220.

[72] C. Cai, H. Zhang, X. Zhong, L. Hou, J. Hazard. Mater. 283 (2015) 70-79.

[73] S.O. Ganiyu, T.X. Huong Le, M. Bechelany, G. Esposito, E.D. van Hullebusch, M.A. Oturan, M. Cretin, J. Mater. Chem. A 5 (2017) 3655-3666.

[74] Z. Jia, X. Duan, P. Qin, W. Zhang, W. Wang, C. Yang, H. Sun, S. Wang, L.-C. Zhang, Adv. Funct. Mater. 27 (2017) 1702258.

[75] X. Qin, Z. Li, Z. Zhu, H. Fu, H. Li, A. Wang, H. Zhang, H. Zhang, J. Mater. Sci. Technol. 34 (2018) 2290-2296.

[76] Z. Jia, X.G. Duan, W.C. Zhang, W.M. Wang, H.Q. Sun, S.B. Wang, L.C. Zhang, Sci. Rep. 6 (2016) 38520.

[77] P. Wang, X. Bian, Y. Li, Chin. Sci. Bull. 57 (2012) 33-40.

[78] X. Qin, Z. Li, Z. Zhu, H. Fu, H. Li, A. Wang, H. Zhang, H. Zhang, J. Mater. Sci. Technol. 33 (2017) 1147-1152.

[79] P. Liu, J.L. Zhang, M.Q. Zha, C.H. Shek, ACS Appl. Mater. Interfaces 6 (2014) 5500-5505.

[80] L. Ji, J.W. Chen, Z.G. Zheng, Z.G. Qiu, S.Y. Peng, S.H. Zhou, D.C. Zeng, J. Phys. Chem. Solids 145 (2020) 109546.

[81] C. Zhang, H. Zhang, M. Lv, Z. Hu, J. Non-Cryst. Solids 356 (2010) 1703-1706.

[82]L. Hou, Q. Wang, X. Fan, F. Miao, W. Yang, B. Shen, New J. Chem. 43 (2019) 6126-6135.

[83] J.J. Si, J.L. Gu, H.W. Luan, X.L. Yang, L.X. Shi, Y. Shao, K.F. Yao, J. Hazard. Mater. 388 (2020) 122043.

[84] B. Zhao, Z. Zhu, X.D. Qin, Z. Li, H. Zhang, J. Mater. Sci. Technol. 46 (2020) 88-97.

[85] X. Luo, R. Li, J. Zong, Y. Zhang, H. Li, T. Zhang, Appl. Surf. Sci. 305 (2014) 314-320.

[86] S. Nam, P.G. Tratnyek, Water Res. 34 (2000) 1837-1845.

[87]Y. Mu, H.Q. Yu, S.J. Zhang, J.C. Zheng, J. Chem. Technol. Biotechnol. 79 (2004) 1429-1431.

[88] S.Q. Chen, G.N. Yang, S.T. Luo, S.J. Yin, J.L. Jia, Z. Li, S.H. Gao, Y. Shao, K.F. Yao, J. Mater. Chem. A 5 (2017) 14230-14240.

[89] S.B. Hammouda, F. Fourcade, A. Assadi, I. Soutrel, N. adhoum, A. Amrane, L. Monser, Appl. Catal., B 182 (2016) 47-58.

[90] M. AboliGhasemabadi, W. Ben Mbarek, A. Cerrillo-Gil, H. Roca-Bisbe, O. Casabella, P. Blanquez, E. Pineda, L. Escoda, J.J. Sunol, J. Environ. Manage. 258 (2020) 110012.

[91] P.V. Nidheesh, R. Gandhimathi, S. Velmathi, N.S. Sanjini, RSC Adv. 4 (2014) 5698.

[92] P. Nazari, S.R. Setayesh, Int. J. Environ. Sci. Technol. 16 (2018) 6329-6346.

[93] B. Kakavandi, A.A. Babaei, RSC Adv. 6 (2016) 84999-85011.

Acknowledgments

This work was financially supported by the key research and development program of China (Grant No. 2022YFB2404102), the National Natural Science Foundation of China (51971093, 52171158 and 52101196), the Open Project Program of Shandong Marine Aerospace Equipment Technological Innovation Center (Ludong University) (Grant No. MAETIC2021-11) and the key research and development program of Shandong Province (Grant No. 2021ZLGX01 and 2022CXGC020308)
